# Personalized Volumetric Tissue Generation by Enhancing Multiscale Mass Transport through 3D Printed Scaffolds in Perfused Bioreactors

**DOI:** 10.1002/adhm.202200454

**Published:** 2022-07-08

**Authors:** David P Forrestal, Mark C Allenby, Benjamin Simpson, Travis J Klein, Maria A Woodruff

**Affiliations:** ^1^ Centre for Biomedical Technologies Queensland University of Technology 60 Musk Avenue Kelvin Grove Queensland 4059 Australia; ^2^ Herston Biofabrication Institute Metro North Hospital and Health Service 7 Butterfield St Herston Queensland 4029 Australia; ^3^ School of Mechanical and Mining Engineering The University of Queensland Staff House Rd St Lucia Queensland 4072 Australia; ^4^ School of Chemical Engineering University of Queensland Staff House Rd St Lucia Queensland 4072 Australia; ^5^ School of Science and Technology Nottingham Trent University Clifton Campus Rd Nottingham NG11 8NF UK

**Keywords:** 3D printing, bioreactors, computational fluid dynamics, mass transport

## Abstract

Engineered tissues provide an alternative to graft material, circumventing the use of donor tissue such as autografts or allografts and non‐physiological synthetic implants. However, their lack of vasculature limits the growth of volumetric tissue more than several millimeters thick which limits their success post‐implantation. Perfused bioreactors enhance nutrient mass transport inside lab‐grown tissue but remain poorly customizable to support the culture of personalized implants. Here, a multiscale framework of computational fluid dynamics (CFD), additive manufacturing, and a perfusion bioreactor system are presented to engineer personalized volumetric tissue in the laboratory. First, microscale 3D printed scaffold pore geometries are designed and 3D printed to characterize media perfusion through CFD and experimental fluid testing rigs. Then, perfusion bioreactors are custom‐designed to combine 3D printed scaffolds with flow‐focusing inserts in patient‐specific shapes as simulated using macroscale CFD. Finally, these computationally optimized bioreactor‐scaffold assemblies are additively manufactured and cultured with pre‐osteoblast cells for 7, 20, and 24 days to achieve tissue growth in the shape of human calcaneus bones of 13 mL volume and 1 cm thickness. This framework enables an intelligent model‐based design of 3D printed scaffolds and perfusion bioreactors which enhances nutrient transport for long‐term volumetric tissue growth in personalized implant shapes. The novel methods described here are readily applicable for use with different cell types, biomaterials, and scaffold microstructures to research therapeutic solutions for a wide range of tissues.

## Graphical abstract

A biomanufacturing framework achieving personalized and volumetric lab‐grown tissue through additive manufacturing, multiscale computational fluid dynamics, and long‐term perfusion bioreactor cultivation is presented. This framework can be applied to diverse defect shapes, 3D printed scaffold patterns, and bioreactor perfusion strategies to minimize transport limitations and accomplish 60‐fold cell expansion across 3 weeks of culture in 13 mL‐volume, 10 mm‐thick scaffolds.

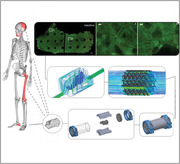

## 1 Introduction

Tissue defects have unique anatomical shapes arising from trauma, congenital abnormalities, and surgical interventions. Graft material sourced from donor tissue is limited in quantity, shape, and microstructure and carries risk of infection. Custom‐designed acellular implants are used to treat tissue defects but demonstrate poor regenerative capacity.^[^ ^1^ ^]^ Pre‐cellularized implants enable the regeneration of tissue across larger volumetric defects but remain limited due to ineffective nutrient transport to support the cells.^[^ ^2^ ^]^ The ideal implant for large defect regeneration would be fabricated in a patient‐specific shape, incorporate high cell densities, and enable sufficient nutrient‐exchange to survive long‐term.^[^ ^3^ ^]^ Laboratory bioreactors capable of regenerating personalized large tissue defects are required in order to understand how custom‐shaped volumetric tissues can be achieved, controlled, optimized, and manufactured to enable future therapeutic use of cellularized implants.^[^ ^4^ ^]^

Additive manufacturing equipment capable of forming biomaterials into personalized engineered tissue substitutes is now well‐established.^[^ ^5^, ^6^ ^]^ Multiple materials, varying microstructures, and live cells within bioinks can be deposited using robotically controlled fabrication techniques such as 3D printing. This allows unique one‐off constructs to be produced using standardized equipment.^[^ ^7^, ^8^, ^9^ ^]^ A key benefit of these systems is the ability to take patient medical scans and rapidly manufacture a personalized implant, followed by the ability to rapidly produce a subsequent patient's implant, with different implant anatomy without the need for costly and time‐consuming changes to machine tooling, equipment, and quality assurance processes. This semi‐automated customization provides considerable benefits within the medical field as each patient and defect site has unique requirements that benefit from tailored treatments.^[^ ^10^ ^]^ While additive manufacturing enables the patient‐specific placement of cells and materials within implants, challenges in mass transport limit the supply of essential nutrients to cells within large 3D scaffolds. This results in scaffolds with only a thin shell of viable cells on the periphery and a necrotic core of dead cells in the center, beyond a few hundred microns of depth.^[^ ^4^, ^11^, ^12^, ^13^, ^14^ ^]^

Perfusion bioreactors are dynamic cell culture systems that enable the laboratory growth of volumetric tissues. These bioreactors aim to enhance the transport of culture media throughout the pores of the cellularized scaffold in a controlled distribution to ensure essential nutrients, dissolved gasses, and hydrodynamic forces are delivered appropriately to cells throughout the full scaffold volume.^[^ ^15^, ^16^, ^17^, ^18^, ^19^, ^20^, ^21^ ^]^ Design is relatively straightforward for simple scaffold shapes such as a cylinder with a uniform cross‐section; however, complex 3D printed lattice scaffolds manufactured in anatomical shapes cause difficulties at multiple scales. At the microscale, the laydown pattern of 3D printed fibers may cause an inconsistent distribution of pores, leading to variability in the distribution of flow, corresponding to non‐uniform nutrient supply and variable hydrodynamic shear forces applied to cells in different regions of the scaffold.^[^ ^15^, ^22^, ^23^ ^]^ At the macroscale, anatomically shaped scaffolds cause difficulties in designing close‐fitting chambers or inserts that force flow to perfuse through the scaffold.^[^ ^4^ ^]^ Consequently, perfused bioreactor cultures of cellularized implants remain poorly applied to patient‐specific manufacturing as a new scaffold and perfused bioreactor designs would be required for each patient's defect which would be cost and time prohibitive.

The successful growth of tissue throughout volumetric and anatomically shaped scaffolds has only been accomplished in a limited number of studies, and none, to our knowledge have aimed to provide rapid and inexpensive means of culturing different scaffold geometries. In some cases, mass transit limitations have been overcome by forcing a flow of culture media using a custom‐designed chamber tightly conforming to the external shape of the scaffold.^[^ ^24^, ^25^, ^26^ ^]^ Perfused medium flow has been better homogenized by surrounding the scaffolds with a porous diffuser of similar permeability but difficulties in closely conforming the diffuser to the scaffold shape persist as well as challenges in separating the regenerated tissue scaffold from the porous diffuser at the end of culture.^[^ ^27^, ^28^, ^29^ ^]^ Scaffolds can also incorporate hollow channels to better distribute medium deep within the center of the engineered tissue, but this is often to the detriment of mechanical integrity and tissue volume.^[^ ^30^, ^31^, ^32^ ^]^ Although these studies propose innovative solutions to the challenges of mass transit, these systems require considerable analysis, redesign, and remanufacturing before they can be applied to a different scaffold with bespoke anatomical shapes or microstructures. Furthermore, no studies have compared these anatomical scaffold perfusion techniques directly against each other and against static controls.

**Figure 1:**
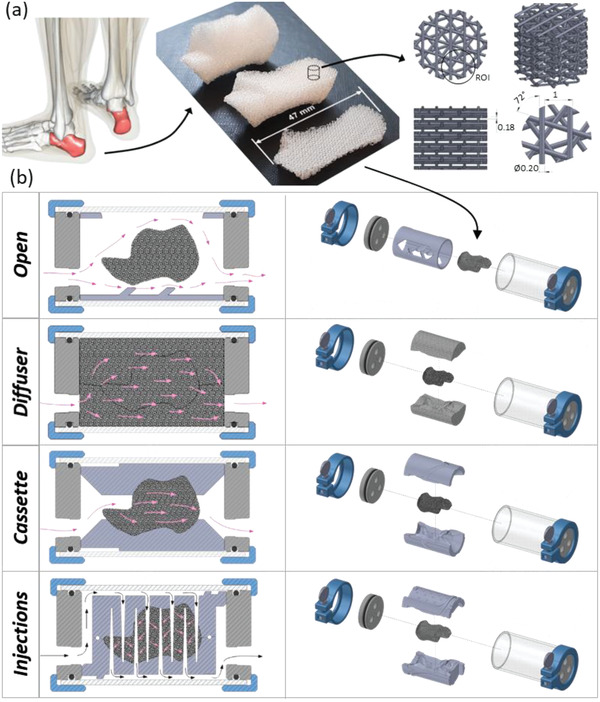
The universal design of 3D printed scaffolds and chamber inserts to control the transport of perfused cell culture medium. a) Using an example human calcaneus (heel bone) and optimized 3D printed structures, tissue scaffolds were designed to fit within a universal bioreactor chamber using flow‐controlling inserts which could be easily assembled to the scaffold, complete enclosing it and conforming to its external shape. b) Four different flow‐focussing inserts were designed and compared against each other and static controls during CFD simulations and 7‐day, 20‐day, and 24‐day bioreactor cell culture. On the left, a cross‐sectional view of bioreactor designs illustrates the flow of medium using pink arrows. On the right, an exploded CAD rendering illustrates how the scaffold, inserts, and bioreactor shell were assembled.

In this work, we present a framework to overcome these remaining challenges in the use of additive manufacturing for volumetric and personalized perfusion tissue bioreactors. Our approach involves simulating and experimentally validating the microscale fluid flow and permeability created by perfusing culture media through different 3D printed fiber lay‐down patterns in scaffolds. The determined permeability of these 3D printing patterns was fed into automated design tools for the rapid creation of bioreactor inserts and scaffolds for a variety of bone anatomies. Four different bioreactor perfusion modalities were computationally and experimentally compared by assessing their efficacy to grow volumetrically‐confluent and homogenously‐distributed tissue in a 13 cm^3^ human calcaneus scaffold shape across 24 days. While open chamber perfusion of unfocussed flow only enabled tissue growth near the scaffold surface, flow‐focussing diffuser, cassette, and injections bioreactor designs illustrated in **Figure** **1** were able to extend tissue growth throughout the remaining 80% of the scaffold volume to its full depth of 1 cm. A critical benefit of the biomanufacturing framework developed here is its ability to be optimized for the production of various cellularized scaffold size and shape using one standard bioreactor chamber illustrated in Figures S1–S3, Supporting Information. This framework addresses a key challenge of current culture systems and cellularized implant manufacturing by combining anatomically precise 3D printing with structural and flow‐optimized cultivation of tissue throughout large and complex volumes.

## 2 Results

### 2.1 Microscale Model‐based Design of 3D Printed Scaffolds Minimize Perfusion Back‐Pressure

An automated computer‐aided design (CAD) and computational fluid dynamics (CFD) model was developed to evaluate medium perfusion through melt‐extruded scaffolds given input parameters of layer height, fiber diameter, angle offset of fiber axis between adjacent layers, fiber spacing, scaffold height, and cylinder diameter (Figure S4, Supporting Information). The purpose of this model was to first allow microscale investigation of the flow through the pores of the scaffold with properties such as local fluid velocity, wall shear stress, and streamlines indicating the path fluid takes as it moves through the structure. The second purpose of this model was to determine the permeability of the porous structure of the scaffold, which could then be applied as a bulk field property of the porous zone of much larger scaffolds. This method avoided the massive computational resource required to resolve the entire complex micro‐porous structure of large anatomical scaffolds in the macro‐scale simulations. Although many tissue engineering studies simulate flow through porous scaffolds and bioreactors, few validate the accuracy of these simulations using experimental flow rig measurements.

**Figure 2:**
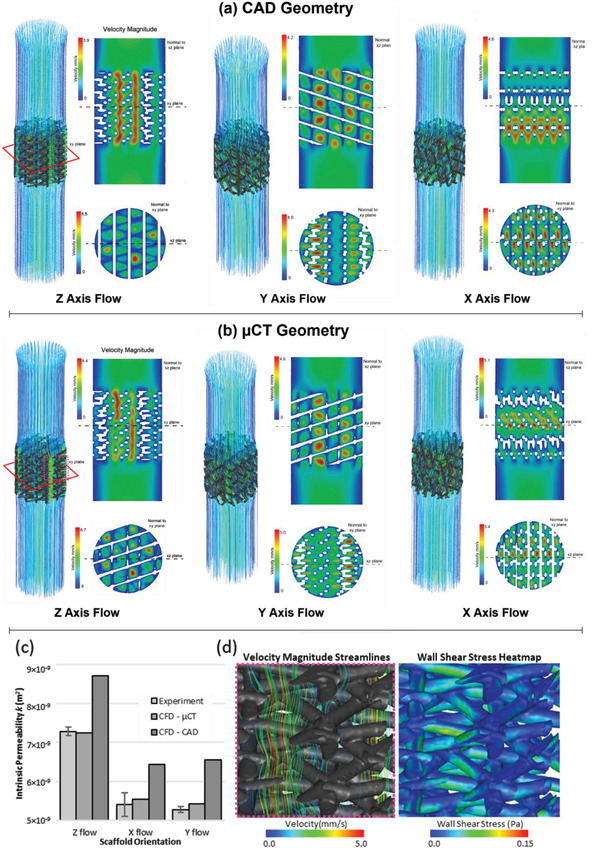
µCT‐based fluid dynamics accurately predict 3D printed scaffold designs with minimal resistance to flow. a) Microscale CFD simulations modeled the flow through ideal CAD scaffold geometries used to design the scaffold. Flow was simulated through X, Y, and Z directions through the scaffold structure with the Z axis perpendicular to the fiber deposition plane of the printed scaffold. Images are colored to show local fluid velocity magnitude. b) Simulations through scaffold geometries reconstructed from µCT images of a 3D printed sample. Patterns of higher local velocities in regions of larger aligned pores, match the behavior shown in the µCT simulation results; however, quantitative differences in velocity magnitude were present between the µCT and CAD samples. c) Intrinsic permeability of the scaffold calculated experimentally compared with results from the CFD simulations using the parametric CAD model and also the µCT scaffold geometry. Error bars show standard deviation for experimentally derived constants (x flow n = 7, y flow n = 4, and z flow n = 7). d) Fluid flow through µCT CFD models occurred predominately between aligned pore gaps commensurate with an increase in wall shear stress (images represent 2.5 mm scaffold width). Simulations performed using water with laminar, steady‐state conditions.

Simulations of fluid flow were performed through Ø 5 mm x 5 mm cylindrical ROIs for both CAD and µCT‐derived scaffold geometries at an average fluid velocity of 1.18 mm s^−1^. Flow through X, Y, and Z directions was simulated, where z was perpendicular to the scaffold deposition plane during 3D printing (Figure S5, Supporting Information). Scaffolds were approximately 37% less restrictive to flow in the direction tangential to printing (Z), with a mean intrinsic permeability of 7.30 × 10^−9^ m^2^ in the Z axis as compared to 5.40 × 10^−9^ and 5.27 × 10^−9^ m^2^ in X and Y directions, respectively (**Figure** **2**c). No statistical difference was found between X and Y direction results, which were averaged to a value of 5.34 × 10^−9^ m^2^ and used in bioreactor CFD model‐based design. CFD simulations revealed scaffold pores aligned in the Z direction created larger continuous channels compared to more tortuous flow paths in the X and Y directions. This created regions of higher velocity fluid flow which were responsible for the lower permeability of the scaffold in the Z direction (Figure 2a, b). These simulation results were then validated against experimental flow rig measurements using 3D printed scaffold samples.

**Figure 3:**
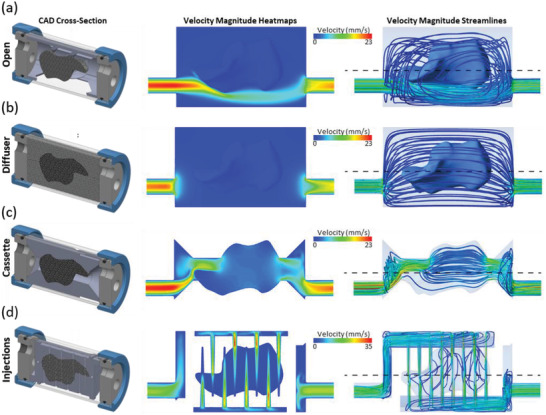
Design and simulation of bioreactor fluid dynamics to predict scaffold mass transport. a–d) Design rendering and CFD simulation velocity heatmaps and streamlines are shown for four flow‐focussing designs. (a) The open chamber design, (b) The diffuser design, (c) the cassette design, and (d) the injections design as illustrated through 3D renders alongside CFD velocity heatmaps and streamlines.

Experimental measurements of permeability were nearly identical to µCT CFD simulations (less than 3% difference), but significantly different from the parametric CAD model CFD simulations (a difference of 18.9% X flow, 24.7% Y flow, 19.1% Z flow). This was due to small differences in fiber placement and morphology of the printed samples compared to the “ideal” CAD geometry (Figure 2c). Surface deviation analysis of CAD versus µCT models showed a 0.05 mm average surface deviation (Figure S6, Supporting Information). Both CAD and µCT models predicted similar general flow behavior with regions of high wall shear stress within aligned porous gaps between fiber layers, creating flow channels with high fluid velocities (Figure 2d). Wall shear stress on scaffold surfaces was shown to range between 0–0.15 Pa (Figure 2d; right image) corresponding to local fluid velocities within the pores of 0–5 mm s^−1^. This spatial pattern of shear stress and local velocity within the pores arose from an average fluid velocity of 1.18 mm s^−1^ through the scaffold structure. Shear stress magnitude is expected to scale with variation seen in the average fluid velocity through different regions of the full‐sized scaffolds (**Figure** **3**); however, it is likely that the pattern of shear stress distribution within the pores is similar.

While biomechanics of 3D printed scaffolds are often simulated using idealized CAD models to reduce computational complexity,^[^ ^33^, ^34^, ^35^, ^36^, ^37^ ^]^ our results indicate microscale 3D printing defects create substantial changes to scaffold permeabilities which are important to design perfused bioreactor mass transport.^[^ ^38^, ^39^ ^]^

### 2.2 Macroscale Modular Bioreactors Integrate Patient‐Specific Anatomy and Enhance Nutrient Perfusion

Leveraging microscale scaffold CFD simulations and experiments, four bioreactor culture chambers were designed to control the perfusion of culture media throughout large anatomically shaped scaffolds using the calcaneus (heel) bone as an example anatomy. Scaffold permeability determined from the microscale study was applied to all scaffolds and porous diffuser components. Flow rate was set to 40 mL min^−1^ corresponding to an average fluid velocity of 1.18 mm s^−1^ through the scaffold to align with the conditions of the microscale study. Viscosity (0.76 mPa s) and density (996 kg m^−3^) of culture media at 37 °C were experimentally measured and applied as fluid properties for the simulations (Figure S7, Supporting Information).

The open chamber bioreactor simply supports the scaffold centrally within the chamber but did not include any method of forcing perfused medium to flow through the body of the scaffold. Simulation results showed perfused medium flows from the inlet around the outside of the scaffold through the chamber to the outlet. Regions of fluid stasis occur within the scaffold, with velocity vectors randomly oriented within the scaffold indicative of no significant flow in any direction. The bulk of flow is shown to take the most direct path from the inlet to the outlet, in almost a straight line except for obstructions caused by the scaffold which force the jet of flow to go around the outside (Figure 3a, Figure S8, Supporting Information).

The diffuser bioreactor encases the scaffold within holders of equal porosity and permeability to distribute the flow evenly throughout the bioreactor chamber. Fluid velocities of 0.5 mm s^−1^ were consistent throughout the scaffold, with entrance and exit effects creating slightly higher velocities of 0.8 mm s^−1^ at the scaffold's ends (Figure S9, Supporting Information). While velocity vectors show a uniform direction of flow through the chamber, the scaffold comprises only a fraction of the total flow space so that flow into the scaffold is lower than total bioreactor flow (Figure 3b). The method is shown to homogenize and distribute the flow, but not all the flow goes through the body of the scaffold.

The cassette bioreactor forces all medium flow through an angled, narrow channel to the front face of the scaffold. The channel closely conforms to the scaffold shape ensuring all flow is forced to go through the scaffold. However, flow preferentially travels through the shortest distance through scaffold, neglecting the scaffold's top and bottom. Static regions are present away from the central flow path, in particular at the upper and lower extremities shown in the XY plane (Figure 3c). Much higher velocities are present within the scaffold, with the section plane showing central regions with 2 mm s^−1^ fluid velocity (Figure S10, Supporting Information).

The injection bioreactor includes a series of hollow channels throughout the scaffold such that fluid is forced to perfuse the scaffold pores from the inlet channels to adjacent outlet channels as it is pumped through the chamber (Figure 3d). Fluid velocities are lower at the entrance of inlet channels at the bottom of the scaffold, and higher at the exit of the outlet channels at the top of the scaffold. While there are high velocities of up to 30 mm s^−1^ within the channels, lower velocities exist throughout cell‐growing porous zones of less than 2 mm s^−1^ (Figure S11, Supporting Information).

### 2.3 Multiscale Geometric Optimization of Perfusion Enables Dense Long‐Term Volumetric Tissue Growth

**Figure 4:**
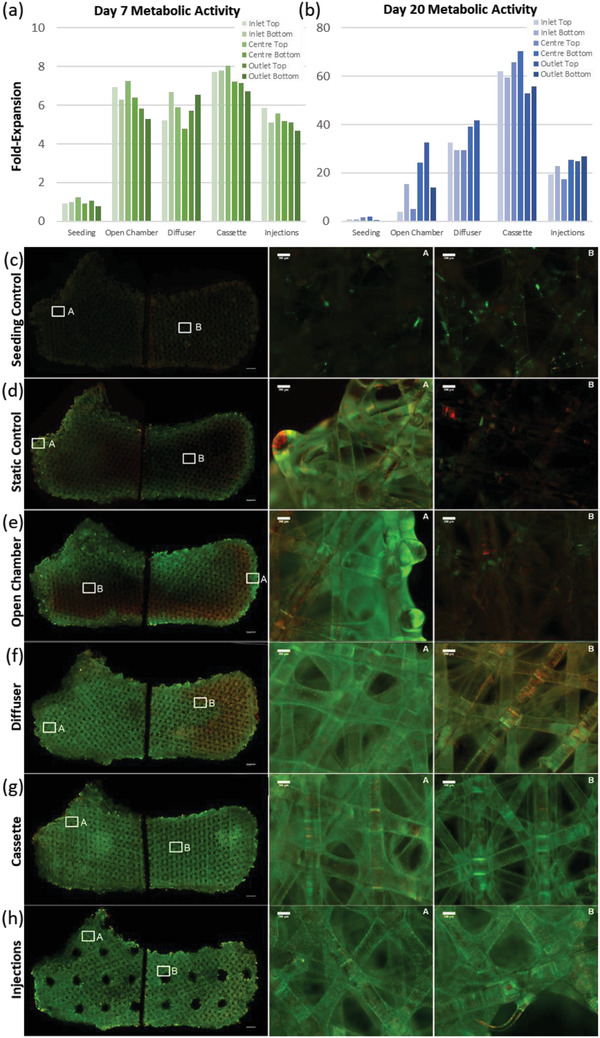
Mass transport limitations in volumetric, long‐term tissue growth can be overcome through flow‐focussing bioreactors. a) Cellular fold‐expansion within scaffold biopsies after 7 days of bioreactor culture, normalized to metabolic activity of day 0 scaffolds seeded with 7 million cells as assessed by Alamar Blue assay. b) Cellular fold‐expansion within scaffold biopsies after 20 days of bioreactor culture, normalized to metabolic activity of day 0 scaffolds seeded with 1.6 million cells. c–h) Images of live and dead cells throughout whole‐bioreactor cross sections at day 20 and annotated regions of interest as stained by Calcein AM in green and ethidium homodimer‐1 in red. Bioreactor cross‐sections were cut in half to fit within an imaging plate. All images are from the same tile scanning sequential image capture on a Zeiss Axio Imager M2 Fluorescent Microscope. ROIs A and B are cropped from larger tile scanned image shown on the left. ROI A and B width 2580 µm. Scale bars of whole‐bioreactor confocal images are 2000 µm (c–h, left column), and scale bars of ROIs are 200 µm (c–h, center and right columns).

The four simulated perfused bioreactor designs and non‐perfused static and seeding controls were manufactured, sterilized, conditioned with medium, seeded with murine pre‐osteoblast cells, then cultured for 7, 20, and 24 days. Cell expansion was assessed using metabolic assays. Within the 7‐day culture, 7 million seeded cells per scaffold underwent a five to sixfold expansion throughout the perfusion bioreactor platforms with the injections bioreactor designs containing slightly fewer cells perhaps due to the presence of hollow channels (**Figure** **4**a). The distribution of cells within the 7‐day bioreactors did not present significant mass transport limitations (Figure S12, Supporting Information). In contrast, the 20‐day bioreactor cultures were seeded with 1.6 million cells per scaffold and reached tenfold (open chamber), 20‐fold (injections), 33‐fold (diffuser), and 60‐fold expansion (cassette). At day 20, clear differences exist between metabolic assays (Figure 4b) and cell distribution (Figure 4c–h) for each bioreactor design.

The seeding bioreactors (scaffolds at day 1) were sectioned, assayed, and imaged after 24 h of static culture post‐seeding with cells. They showed a sparse but regular distribution of seeded cells throughout all cross sections for each experiment (Figure 4c, Figures S13–S16, Supporting Information).

The static bioreactors resulted in well‐defined superficial regions of cell proliferation at day 20 with few viable or dead cells beyond 2–3 mm of the external surface (Figure 4d, Figures S15–S18, Supporting Information). These controls suggest that mass transit limitations beyond the surface occurred rapidly, preventing early cell proliferation that would subsequently result in large numbers of dead cells.

The open chamber bioreactors supported high cell proliferation in the first week of culture, but growth was similarly restricted to superficial regions and plateaued after day 7. In contrast with the static control bioreactor, these superficial regions of tissue growth were much larger in the regions of scaffold where the majority of perfused medium was simulated to travel (Figure S8, Supporting Information). A higher cell death was observed in comparison to static control bioreactors, with a high viability surface zone first transitioning to higher concentrations of dead cells before a fluorescent signal reduction in the central regions indicating few total cells (Figure 4e, Figure S19, Supporting Information).

The diffuser bioreactors enabled sustained cell growth that lasted into the fourth week. The diffuser design achieved a dense growth of tissue throughout the scaffold with a thin superficial layer of confluent tissue (Figure 4f, Figures S15–S18, Supporting Information). Diffuser scaffolds exhibited a pronounced gradient of increased cell death from the flow inlet to the flow outlet of the scaffold, suggesting an inefficient mass transfer unable to overcome nutrient consumption or metabolite production towards the outlet side of the scaffold (Figure 4f, Figure S19, Supporting Information).

The cassette bioreactors achieved the highest proliferation rate at day 20, commensurate with the densest quantity of tissue throughout the volumetric scaffolds. The densest tissue growth remained on the scaffold surface, comprising several millimeters of confluently grown cells. These regions exhibited a thick webbing of confluent cells between PLA fibers, which in some areas extended deep into the scaffold core (Figure 4g, Figure S20, Supporting Information).

The injection bioreactors exhibited a lower metabolic activity at day 20 in comparison with diffuser and cassette scaffolds, however, a high content of thick viable tissue was imaged throughout the scaffold. This discrepancy between metabolic activity and imaging may be attributed to injection scaffolds having a smaller available volume of culture surface area within the pores due to the removal of scaffold material to create perfusion channels. Thick regions of confluent cells with webs between scaffold fibers were present in the immediate vicinity of the injection channels and around some of the scaffold external surfaces. Beyond a few millimeters of distance from the injection channels and scaffold surfaces, regions were present with lower cell densities (Figure 3h, Figure S18, Supporting Information).

**Figure 5:**
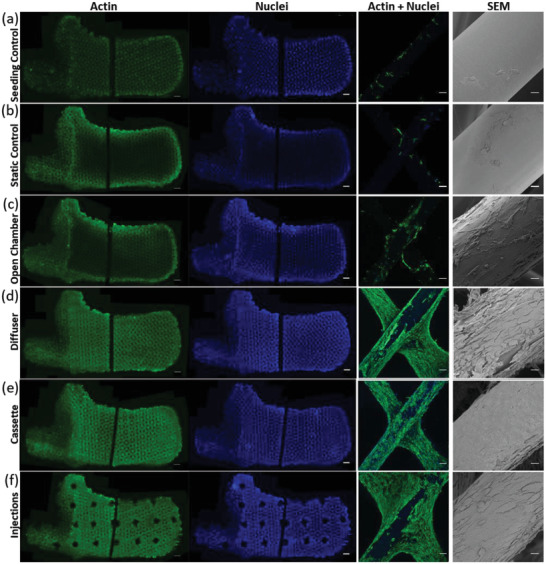
Layers of confluent tissue are achieved for flow‐focussing bioreactor designs. a–f) Images of cell cytoskeleton and nuclei throughout whole‐bioreactor cross sections at day 20 alongside confocal scanning laser microscope images and SEMs at the core of the scaffold. Florescent imaging samples were stained by phalloidin‐conjugated AlexaFluor 488 in green and 4′,6‐diamidino‐2‐phenylindole (DAPI) in blue. The bioreactor scaffold cross‐sections were cut in half to fit within an imaging plate. Scale bars of whole‐bioreactor confocal images are 2000 µm, ROI confocal images are 100 µm, and SEM images are 20 µm.

Reasonably consistent distribution of attached cells within Seeding control scaffolds was shown from spatial sampling of metabolic activity (Figure 4a,b), fluorescent microscopy of cell nuclei (**Figure** **5**a, Figures S12 and S14, Supporting Information), live cells (Figure 4c, Figure S13, Supporting Information) and SEM imaging at the periphery and central scaffold regions (Figures S15, S16, S21, Supporting Information). However, after 20 days of growth, cells in static control and open chamber scaffolds were concentrated near scaffold surfaces, with only sparse distributions of low viability cells in the core (Figures 4c,d and 5b,c). In contrast, the perfused diffuser, cassette, and injections bioreactor designs all show a confluent monolayer or multilayer coating of cells on fibers in the center of the scaffold, with tissue inside the core of the diffuser, cassette, and injections bioreactors noticeably webbing across pores between fibers (Figure 4e,f,g, Figure 5d,e,f, Figures S19 and S20, Supporting Information).

**Figure 6:**
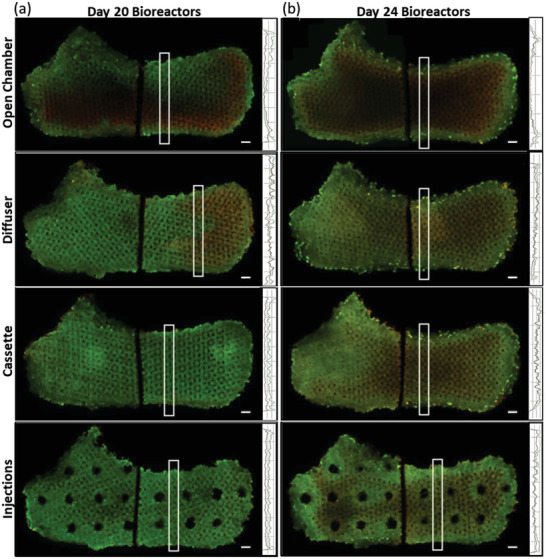
Patterned mass transport limitations from increased cell density and culture duration. A comparison of live and dead cell distribution after a) 20‐day culture of 1.6 million cells per scaffold versus b) 24‐day culture of 3 million cells per scaffold. Images of live and dead cells throughout whole‐bioreactor cross sections were visualized by Calcein AM in green and ethidium homodimer‐1 in red. On the right of each image includes a line graph of each stain's fluorescent intensity averaged over the annotated 1000 µm wide rectangle of interest. The bioreactor scaffold cross‐sections were cut in half to fit within an imaging plate. Scale bars of whole‐bioreactor confocal images are 2000 µm.

To further push the limits of mass transport in our platform, bioreactor cultures were seeded with 3 million cells per scaffold and cultured for 24 days. Significant mass transport limitations were evident by the steep gradients of tissue viability imaged at day 24, when viable cells in superficial zones appeared to be completely filled in with thick tissue, after which larger central regions of intense necrotic tissue existed in comparison to prior day 20 cultures (**Figure** **6**). While mass transport limitations were present within 20‐day cultures, these higher‐density 24‐day cultures emphasize these patterns, including a more pronounced inlet‐to‐outlet gradient of increasing dead cells in the diffuser scaffold, a build‐up of dense living tissue followed by dead tissue from inlet‐to‐center of the cassette scaffold, and clear gradients of dead tissue away from the perfused channels of the injection scaffold. Spatial patterns of cell death did not correspond to regions of the scaffold simulated to have the highest fluid velocity and shear stress applied to cells (Figure 3). Rather, periphery regions of the open chamber, cassette, and injection scaffolds with the highest local fluid velocity exhibited dense populations of viable cells. Consequently, mass transit limitations are the driver of cell death rather than excessive fluid shear stress.

## 3 Discussion

Mass transport remains a critical bottleneck for biomanufacturing patient‐specific tissue constructs. Only small or thin lab‐grown tissues or cell suspensions have received FDA approval for clinical treatments, including blood cells, wrinkle filler, skin grafts, cornea replacements, and thinly‐layered osteochondral or mucosal grafts.^[^ ^40^ ^]^ The biomanufacture of volumetric tissues containing densely packed cells could provide a greater functionality, efficiency, and breadth of lab‐grown grafts. Recent studies have achieved this volumetric tissue growth using perfused medium or hollow channels, but personalized approaches remain largely ad hoc as there remains an incomplete understanding of how multiscale scaffold structure and nutrient perfusion influence mass transport for volumetric tissue growth.

In this study, engineered tissue mass transport has been pursued through two strategies: 1) engineering scaffold‐internal microstructures with predictable medium permeability and 2) designing scaffold‐external bioreactors which enhance medium inflow and can be readily applied to personalized anatomic scaffold shapes. Tissue generation over 5 mm of depth remains challenging,^[^ ^41^ ^]^ yet, here we have demonstrated a manufacturing pipeline that leverages 3D printing of inexpensive scaffolds and bioreactors to generate full‐thickness tissue at 13 cm^3^ volume and over 10 mm depth during long‐term culture in anatomically‐specific shapes. Our pipeline leverages multiscale fluid dynamics simulations and innovative flow‐focussing bioreactor designs to engineer perfusion strategies for homogenous and complete mass transport of nutrients throughout the large scaffolds, thereby providing new strategies toward tissue patterning through large complex scaffolds.

3D printing enables greater control over scaffold permeability and bioreactor mass transport compared to prior studies using porous foam scaffolds and machined bioreactor assemblies. There exist many studies which simulate the fluid dynamics of culture media through perfusion 3D bioreactors to optimize fluid flow, shear rates, or cell culture densities.^[^ ^42^, ^43^, ^44^, ^45^, ^46^, ^47^ ^]^ However, there are few studies that experimentally validate the flow predicted from CFD investigation. In addition, many studies assume one bulk scaffold permeability for CFD studies which would not account for the potential anisotropic flow properties of tissue engineering scaffolds.^[^ ^48^, ^49^ ^]^ Here we sought to address these issues with the construction of an experimental flow testing rig to validate assumptions regarding flow simulations and scaffold permeabilities for different 3D printed scaffold microstructures.

Traditional tissue engineering studies grow cells in a scaffold using static culture, where the scaffold is immersed in culture media which is occasionally refreshed.^[^ ^50^ ^]^ Many tissue engineering bioreactor studies incorporate medium perfusion in a similar manner as our open chamber design, where medium is allowed to flow around the cellularized constructs.^[^ ^51^, ^52^ ^]^ In static and open chamber bioreactors, cell growth is frequently limited to a thin superficial layer on the scaffold surface with limited proliferation and cell death in the core.^[^ ^25^, ^53^, ^54^ ^]^

In our diffuser bioreactor design, we filled the entire chamber with a porous material of identical permeability to the scaffold, to homogenize flow and prevent the medium from flowing preferentially around the outside of the scaffold. The diffuser design was successful in achieving viable cell culture conditions within the thickest regions of the scaffold; however, a transition was present towards a zone of low viability at one end of the scaffold corresponding to the outlet side of the chamber. This could be due to a lower effective flow of media through the scaffold, compared to the cassette and injection designs at the same bioreactor volumetric flow rate, since some of the flow proceeds through the porous diffuser around the outside of the scaffold. The use of additional porous geometry within perfusion chambers to homogenize the flow of culture media through 3D printed tissue scaffolds has previously been investigated, but no studies combine CFD predictions with cell culture.^[^ ^27^, ^28^ ^]^ In addition, previous diffuser bioreactor designs present difficulties in removing tissue scaffolds from the diffuser supports after culture,^[^ ^28^ ^]^ or hold the scaffold in a separate mold creating fluid resistance between the scaffold and diffuser.^[^ ^29^ ^]^

Cassette bioreactors have previously been applied to focus the flow of medium directly into cellularized scaffolds. Prior cassette bioreactors include a decellularized bovine trabecular bone CNC‐machined into the shape of human temporomandibular joint condyle (TMJ), seeded with hMSCs, and cultured within a custom‐designed chamber molded from flexible polydimethylsiloxane around a machined polyoxymethylene dummy part in the exact shape of the TMJ scaffold.^[^ ^24^ ^]^ Similar silicones have often been leveraged as they can stretch to provide an air‐tight fit around a scaffold,^[^ ^15^, ^25^ ^]^ but this approach is limited by not being universal to a range of scaffold sizes and shapes and requires a bespoke bioreactor to be designed and manufactured for each new shape. The cassette methodology used in this study is advantageous as different size and shape scaffolds can readily be cultured in the same bioreactor.

Injections bioreactors deliver flow through engineered microchannels which minimize the distances nutrients are required to diffuse to nourish the entire construct. Channels have previously been incorporated throughout engineered scaffolds with and without perfusion to enhance the transport of nutrients for volumetric tissue growth.^[^ ^26^, ^31^, ^55^, ^56^ ^]^ These channels are the most suitable bioreactor designs for culturing scaffolds with even greater volumes and more complex shapes as local fluid velocities do not need to increase with increased scaffold volume, avoiding the effects of excess shear stress applied to cells. Compared to methods applying channels to large, personalized constructs,^[^ ^26^ ^]^ our method is beneficial as scaffold redesign for different anatomies is readily achieved using Boolean CAD operations such as solid model subtraction, addition, and combination, rather than a complete redesign of the injection array. However, the inclusion of channels reduces the available porous scaffold microstructure for tissue to grow, leading to a reduced maximum tissue volume. Furthermore, channels incorporated into scaffold structures to aid media perfusion affect mechanical performance. For example, Finite Element Analysis (FEA) simulation of the increase in stress resulting from the channels added to the Injections scaffold predicted a 6% increase in stress with loads applied in the same direction as the channels and a 119% increase in stress for loads applied perpendicular to the channels (Figure S22, Supporting Information). Prior to clinical use, scaffold mechanical performance requirements must be defined and considered in addition to permeability and features to improve mass transit.

This research presents a design pipeline to regenerate thick confluent tissue throughout large, patient‐specific scaffolds in a universal bioreactor system. Using additive manufacturing and CFD, this pipeline provides enhanced internal scaffold‐permeability combined with external scaffold bioreactor perfusion strategies to grow 13 cm^3^ of tissue in human calcaneus shapes over 24 days of culture. Although only the calcaneus scaffold was validated with cell culture, this platform allows other patient‐specific scaffolds to be rapidly and inexpensively cultured within this modular bioreactor platform (Figures S1–S3, Supporting Information). Due to the large culture medium requirements of these platforms, only 1 or 2 bioreactor replicates were cultured for each time point in the present study. But these three time points demonstrated consistent patterns of viable tissue growth with respect to mass transport gradients, where mass transport limitations were minimal at day 7 (Figure S12, Supporting Information), considerable at day 20 (Figure 5), and led to necrotic tissue in the core of the scaffolds at day 24 (Figure 6). Future work investigating spatial and temporal variation in cell proliferation could include multiple shorter time points to provide insight into the development of cultured tissues and effects on mass‐transit‐driven viability. The significant tissue growth spanning and blocking scaffold pores at late stages of culture were not considered in the CFD models and could be captured using contrast agents and µCT to develop and validate a simulation model including cell activity.^[^ ^57^ ^]^ This would be valuable for understanding flow changes over time as cells and extracellular matrix develop within the pores, and aid understanding of how dissolved species such as oxygen varies within the cultures.

The cell culture experiments were designed as a targeted proof‐of‐concept to test the capacity of the novel bioreactor systems to support dense populations of viable cells by overcoming mass transit limitations. MC3T3 cells were a good candidate for the initial test of system performance due to their rapid expansion and relative stability during culture. Future applications of these bioreactors should focus on more clinically relevant cells and aim to develop matured tissues by optimization of behaviors including extracellular matrix production, differentiation, and growth factor secretion.

## 4 Conclusion

Engineering lab‐grown tissue implants have the potential to overcome many limitations of existing clinical treatments for challenging problems such as critically sized bone defects and could offer alternatives to autografts and allografts which are limited in material shape, size, and quality to the available donor tissue. The development of large, volumetric, and personalized tissue implants remains limited by transporting sufficient medium to sustain large volumes of densely packed cells in a manner that can be inexpensively and rapidly adapted to each patient defect's shape and size. In this study, a design framework was developed to engineer customized 3D printed scaffolds and perfusion bioreactors that deliver an optimal flow of culture media to cells regardless of scaffold shape, thus overcoming a key limitation in the field and demonstrating a universal platform for culturing diverse patient‐specific scaffolds. This framework was validated on human calcaneus scaffolds which were 13 cm^3^ in volume and 10 mm thick to grow confluent or patterned tissue over long‐term culture, suggesting that this framework is uniquely suited to minimize or strategically leverage mass transport gradients throughout volumetric and patient‐specific tissue shapes.

## 5 Experimental Section

### Scaffold Design and Fabrication

A parametric CAD model of a 3D printed scaffold was generated to allow the rapid creation of geometry for a range of different porous structures. The master parametric model was constructed in Solidworks 3D design software (Dassault Systems, Vélizy‐Villacoublay, France) where several user‐input parameters can be varied to optimize scaffold geometry, such as layer height, fiber diameter, angle offset between adjacent layers, fiber spacing, scaffold height and cylinder diameter, layer height, deposition angle, and fiber spacing. 3D printing slicer software was used to setup the manufacturing file for 3D printing (Makerbot Desktop 3.9.1). Scaffolds and diffuser inserts were manufactured from PLA (polylactic acid) thermoplastic (Natureworks Ingeo 3D850) using a modified Makerbot Replicator 2 × 3D printer. Modifications to the printer consisted of an active cooling fan for improved cooling of extruded filament, a glass build surface 0.2 mm diameter nozzle, extruder filament clamping upgrade with a higher spring force and leverage mechanism, and a polytetrafluoroethylene (PTFE)‐lined barrel to reduce drag in the filament melt transition zone. These improvements were implemented systematically to allow reliable printing of PLA material using a 0.2 mm diameter nozzle.

### Micro‐Computerized Tomography

Micro‐computed tomography (µCT) using X‐ray attenuation (µCT 40, Scanco Medical AG, Switzerland) was performed on PLA scaffolds with a voxel resolution of 15 µm to capture the manufactured scaffold geometry for use in the detailed CFD simulations. Scan data was output as a series of grayscale images which were then imported into Amira segmentation software (Version 6.0, FEI, Hillsboro, USA). A surface was created at the boundary between solid and void regions and then exported as a standard tessellation language (stl) model. The stl surface model was imported to Geomagic Wrap mesh processing software (Version 2015, 3D Systems, Rock Hill, USA) where Ø 5.5 mm x 5 mm high regions of interest were extracted from the same stl model in three orientations of cylinder axis; X, Y, and Z (Figures S4 and S5, Supporting Information). These regions were used to extract scaffold cylinders to result in three individual meshes of the same scaffold suitable for simulating flow through different directions. The Z direction was perpendicular to the fiber deposition plane during printing and the X and Y directions were parallel to the deposition plane. Scaffold X, Y, and Z surface models created in Geomagic Wrap were exported as stl files and used as input models for the CFD simulations. No smoothing or sampling was used from the initial scan and segmentation to the exporting of final stl files to ensure the geometric data was captured as accurately as possible for the CFD simulations.

### Perfusion Rig Assembly

Experimental flow rigs were constructed to measure the intrinsic permeability of the scaffold, confirm the laminar nature of the flow, and to validate the results of the micro‐CFD simulations. The flow rigs consisted of 10 mm internal diameter cylindrical tubes containing the 3D printed porous scaffolds and an outer wall of solid material to guide the flow through the scaffolds. These cylindrical rigs were manufactured in 100 mm lengths for three directions of flow described in previous sections; X, Y, and Z. For testing, samples were assembled into a test chamber along with inlet and outlet flow guides to direct the flow through the porous zone. Pressure was measured by a DUXL01D ultra‐low pressure differential pressure sensor (Honeywell, Morris Plains, USA) with a resolution of 0.25 Pa (0.025 mm H_2_O) and required an LM258P operational amplifier (Texas Instruments, Dallas, USA), MCP3426 16‐Bit analog‐to‐digital converter (ADC) (Microchip Technology Inc., Chandler, USA), and Arduino Uno microcontroller (Arduino, Turin, Italy). The pressure sensor was calibrated for each test using pressure head gauges and a series of different pressure versus flow rate values was also checked to ensure sensor output linearity with increasing pressure.

Density and dynamic viscosity measurements of complete culture media consisting of Alpha Minimum Essential Medium supplemented with 10% (v/v) fetal bovine serum and 1% (v/v) penicillin‐streptomycin (10 000 U mL^−1^) (Gibco, Thermo Fisher Scientific, Massachusetts, United States) was measured at 37 °C to accurately reflect fluid properties during cell culture experiments. Very few CFD studies investigating the flow of culture media through tissue scaffolds have experimentally characterized the fluid properties of the culture media (Table S1, Supporting Information). Viscosity testing was carried out on an Anon Parr MCR 302 Rheometer with CP25‐1, 25 mm diameter, and 1° angle measuring cone. Fluid temperature was maintained at 37 °C during the test. Milli‐Q water (Merck Millipore, Billerica, MA) was used as a test fluid for comparison purposes. Testing was performed at three ranges of shear rates to assess non‐Newtonian (shear rate dependent) behavior; 60–160, 250–350, and 600–700 s^−1^.

### Permeability Measurement

Darcy's law has been used extensively in perfusion culture applications for determining the permeability of the scaffold; however, there has been limited experimental validation that the flow was in the laminar regime for tissue scaffold studies.^[^ ^34^, ^38^, ^39^, ^42^, ^58^ ^]^ The bulk permeability of a porous scaffold can be described by Darcy's law:

(1) kscaffold=QLμAΔPscaffoldifRe=ρVDμ<1000

Where, in the experimental flow rigs, *L* is the length of the scaffold, *A* is the cross‐sectional area of flow (void space), *Q* is the measured flow rate, ∆*P* is the measured pressure drop, and µ is the dynamic fluid viscosity which was measured by an Anton Parr rheometer.^[^ ^59^, ^60^ ^]^ Darcy's law is justified for laminar flow Reynold's numbers (Re) where *ρ* is the density of fluid, *V* is the average velocity, and *D* is the characteristic linear dimension. In the case of porous tissue scaffolds, pore diameter has been used as the characteristic length,^[^ ^39^ ^]^ as well as hydraulic diameter defined as four times the fluid volume divided by the wetted surface area.^[^ ^61^ ^]^ With scaffolds used in this study, each of these two methods will produce substantially different characteristic lengths, and consequently different Reynolds numbers for a given set of conditions. For this study, the characteristic length was defined as the pore diameter between adjacent layers of the scaffold fibers. Since no satisfactory study was found providing critical Reynolds numbers for laminar flow through tissue scaffold structures, experimental flow testing was performed at a range of different Reynolds numbers to verify permeability did not change thus confirming the valid use of Darcy's law. Reynolds numbers under 2.5 demonstrated laminar behavior with constant permeability, while Reynolds numbers above 2.5 exhibited a small drift in permeability (Figure S23, Supporting Information). The average velocity used in X, Y, and Z flow CFD simulations results in a Reynolds number of 0.96 indicating that the use of Darcy's law and a laminar flow model was appropriate.

### Microscale Computational Fluid Dynamics

Microscale CFD simulations were performed on scaffold geometries in X, Y, and Z flow directions for both parametric CAD models and reconstructed geometry from µCT data. In both instances, a 5.5 mm diameter by 5 mm high cylindrical ROI of each scaffold was used in CFD simulations. Automated scripts were created to allow the complete process of fluid volume creation, meshing, solving, and results export to be run through a high‐performance computing cluster (SGI Altix XE cluster) using 240 gigabytes of memory and 48 × 2.5 GHz, 64 bit processing cores (E5‐2650v3 64 bit Intel Xeon processor) for each individual simulation. The script imported the stl file containing the scaffold geometry to Fluent CFD simulation software (Version 17.2, ANSYS, Canonsburg, USA) for mesh generation, then created a 26 mm long and 5 mm diameter cylinder to represent the fluid channel.

Within Fluent CFD software (Fluent Meshing Component), a surface mesh was wrapped around the 5 mm diameter fluid channel cylinder and scaffold geometries to produce a conformal mesh representing the fluid volume surface. The scaffold region of interest in the analysis was 5 mm diameter, rather than 5.5 mm, to ensure the scaffold and flow channel intersects for successful wrapping of both surfaces. An inflation layer of volume elements was grown into the fluid volume from the surface mesh, and then the remaining volume mesh was created. Mesh quality statistics including skewness and aspect ratio were automatically examined and automatic re‐meshing was applied where necessary in an iterative process until an acceptable quality mesh was finalized. Automatic mesh improvements were set according to Fluent Meshing recommended settings. Completed meshes were transferred to Fluent CFD Software Component, material properties and boundary conditions were defined along with the laminar flow calculation model. An average inlet velocity of 1.18 mm s^−1^ and zero pressure at the outlet were applied. The solution was then run until convergence of continuity, x, y, and z residuals to 1 × 10^−5^. A surface monitor of pressure drop on the inlet was also included to check solution convergence. A mesh independence study was performed and is presented in Figure S24, Supporting Information.

Fluid velocity boundary conditions were estimated from the previous bioreactor volumetric flow rates normalized to cell number and culture surface area (Table S2, Supporting Information).^[^ ^19^, ^24^, ^62^, ^63^, ^64^, ^65^ ^]^ The µCT model indicated the scaffold had an average of 3.95 mm^2^ of surface area per 1 mm^3^ volume. An average of 6 prior perfused bioreactor studies indicated a flow rate of 0.78 mL min^−1^ per million cells was typical, which provided a calcaneus scaffold flow rate of 40 mL min^−1^ in the bioreactor (assuming confluent cells on scaffold surfaces at a density of 1000 cells mm^−2^). This volumetric flow rate corresponded to an average fluid velocity of 1.18 mm s^−1^, estimated by modeling a constant cross‐section flow channel with an external dimension conforming to the outer shape of the calcaneus scaffold.

### Macroscale Computational Fluid Dynamics

Complete CAD models of bioreactor chamber assemblies with scaffolds and inserts were used to create a model of the fluid zone within the entire bioreactor chamber. 3D data for calcaneus scaffold shape was retrieved from the BodyParts3D database and scaled to 13 cm^3^ volume.^[^ ^66^ ^]^ Porous fluid zones consisting of the calcaneus scaffolds and diffuser inserts were defined within the fluid zone as separate regions with permeability properties determined from the microscale study. Fluent CFD software was used to perform geometry pre‐processing, mesh creation, and fluid dynamics simulation. An automated script was programmed to allow the complete process of mesh creation, simulation, and results export to be performed remotely on the high‐performance computing cluster as described previously for the microscale CFD studies. The script imported the fluid zone geometry, generated the discretized mesh of the fluid volume, and automatically refined and re‐meshed areas of poor mesh quality. Bulk permeability properties were applied to the regions corresponding to scaffold and diffuser insert components as determined during the experimental flow testing and microscale CFD (Table S3, Supporting Information).

Rheometry measurements described in the experimental flow testing determined the mean dynamic viscosity of the culture media used in this study; measured to be 0.76 mPa s at 37 °C compared to a water viscosity of 0.68 mPa s, as shown in Figure S7, Supporting Information. Density of 996 kg m^−3^ and dynamic viscosity of 0.76 mPa s were assigned to the CFD fluid model. The laminar flow calculation model was used with steady state flow and solutions were run till convergence of X, Y, and Z residuals below 1 × 10^−5^. A monitor of pressure drop from bioreactor inlet to outlet surfaces was also included to check solution convergence. A mesh independence study was performed on each chamber design (Table S4, Figure S25, Supporting Information).

### Bioreactor Platform Construction

Culture chamber inserts for the cassette, injections, and the open chamber scaffold holder were manufactured as solid, non‐porous components while scaffolds and the diffuser holder were manufactured as porous components (Figure S26, Supporting Information). Scaffolds and inserts were printed from PLA using the modified Makerbot Replicator 2 × 3D printer previously mentioned. The porous structure of the scaffolds and diffuser inserts consists of consecutively stacked layers of parallel 0.2 mm diameter fibers spaced at 1 mm intervals. Layers were printed at 0.18 mm Z spacing resulting in 0.01 mm of overlap between layer fibers. This overlap was where the fibers weld together to form an interconnecting structure. A 72° rotational offset was applied between layers, resulting in a repeating pattern every 5 layers and scaffold porosity of 77.5%.

Culture chambers were manufactured with borosilicate glass tubes, PTFE end caps, and fluoroelastomer (FKM) seals. Connection tubing consisted of platinum cured silicone, peristaltic pump tubing was PharMed thermoplastic elastomer (BPT) material (Saint‐Gobain, Courbevoie, France), and connection fittings were polyvinylidene fluoride (PVDF). Fluid reservoirs consisted of 80, 100 mL wide mouth borosilicate glass bottles (Duran, Mainz, Germany) and gas exchange was achieved through 0.2 µm filters (Sarstedt, Numbrecht, Germany). Bioreactor chambers, reservoirs, and tubing were held in a custom‐designed laser‐cut acrylic mounting system. The mounting system's modular series of chamber and tube holders held the chambers in a vertical orientation with fluid pumped upwards to prevent air entrapment within the scaffolds. All components in contact with culture media were autoclave sterilized after pre‐assembly to allow minimal handling when loading with cell‐seeded scaffolds and culture chambers.

There were two bioreactor flow platforms: a 7‐day bioreactor platform to test feasibility and a 20‐ or 24‐day bioreactor platform to demonstrate long‐term tissue patterning. The 7‐day platform allowed for a single channel peristaltic pump to supply flow through four culture chambers simultaneously in a parallel arrangement by recycling culture media to a reservoir located above the rest of the system and directing flow through all chambers by flow regulator valves. The long‐term platform (20 and 24 days) connected each bioreactor to an individual flow loop and reservoir with individual peristaltic pump channels using a 12‐channel BVP pump with CA12 head (Ismatec, Switzerland).

### Bioreactor Cell Culture

Scaffolds and inserts were sterilized with a 10‐min immersion in 70% ethanol followed by drying in a biosafety cabinet under UV irradiation. The hydrophobic PLA scaffolds were again pre‐wet with 70% ethanol and then washed with Milli‐Q water (Merck Millipore, Billerica, MA). The water was drained and scaffolds were pre‐conditioned in complete culture media for 2 days prior to cell seeding. MC3T3‐E1 murine precursor osteoblast cells at passage 14 were used for seeding of all scaffolds. Cells were cultured in Alpha Minimum Essential Medium (*α*‐MEM) supplemented with 10% (v/v) fetal bovine serum (Gibco) and 1% (v/v) penicillin‐streptomycin (10 000 U mL^−1^) (Gibco). Environmental conditions were maintained at 37 °C, 98–100% humidity, and 5% CO_2_. Six calcaneus‐shaped scaffold samples with geometry described in previous sections were cultured in the experiment. Two samples were used to assess seeding and attachment, and one sample was used for each 40 mL min^−1^ perfusion culture method consisting of the open chamber, cassette, diffuser, and injection designs.

### Short Term Bioreactor Culture

Scaffolds previously immersed in culture media were stored in individual, sterile, 50 mL falcon tubes. Prior to seeding, culture media was aspirated from the scaffolds and falcon tubes. A suspension of 7.6 million cells in 10 mL of culture media was gently pipetted into each scaffold followed by carefully adding 25 mL of culture media to completely immerse the scaffolds. After seeding, samples were cultured statically for 12 h without removal from the 50 mL falcon tube. Tube caps were loosened allowing gas exchange with the incubator environment. At this point, two samples were removed and processed to investigate cell attachment. Additional cell‐free control scaffolds went through identical preparation and processing steps without the addition of cells.

Scaffolds were removed from static culture 24 h after seeding and assembled with their respective inserts before being loaded into their respective perfusion chambers. The open chamber scaffold was placed directly into the scaffold holder (Figure 1b) before loading into the chamber. For the diffuser scaffold, the inserts were briefly immersed in 70% ethanol, and then thoroughly washed with sterile phosphate buffered saline (PBS) before assembly to the scaffold and placement in the chamber. This pre‐treatment allowed culture media ingress and prevented the entrapment of air bubbles within the insert porosity. Both the cassette and injection inserts required that culture media flow thorough the designed channels for successful perfusion of the scaffolds. To prevent flow leaking through small gaps between the assembled inserts and between the inserts and chamber wall, a system of sealing gel delivery channels was designed into the inserts. This guided the injection of sterilized 5% agarose gel (Roche, Basel, Switzerland) to form a seal. Agarose gel was first prepared by microwave and then sterilized with autoclaving. First, the inserts and scaffolds were assembled and placed into the chamber. The gel was then pre‐heated until completely liquid and injected into the channels. When cooled below 36 °C re‐gelling and formation of a seal occurred. The 4 bioreactor systems contained 500 mL of media each, which was perfused at 28 mL min^−1^ on day 1 and then increased to 40 mL min^−1^ on day 3. Recycled media was changed on day 4 of perfusion with 500 mL of fresh media added (detailed in Table S5, Supporting Information). Scaffolds were analyzed on day 7.

### Long Term Bioreactor Culture

Scaffolds conditioned in culture media were seeded with 3 million cells (Group 1) or 1.2 million cells (Group 2) suspended in 10 mL of culture media as aforementioned. Samples from each group were removed from static culture with and without cells after 24 h and analyzed to provide cell‐free and seeding controls. Scaffolds were removed from static culture 48 h after seeding and placed into assemblies with their respective inserts before being loaded into perfusion chambers. The bioreactor system and chamber inserts were sterilized, washed, and assembled around scaffolds identically, and a sterilized 5% agarose gel was applied for cassette and injection bioreactors to prevent leakage around the internal insert components as aforementioned.

Each bioreactor chamber was connected to an individual flow loop after being loaded with scaffold and inserts. Flow loops were filled with 120 mL of culture media for each bioreactor and the entire system was placed into an incubator. Perfused flow was turned on 24 h after the bioreactor was placed in the incubator and the flow rate was periodically increased from 14 mL min^−1^ on the first day of perfusion to 40 mL min^−1^ at the end of the experiment (detailed in Table S6, Supporting Information). Culture media was changed every 6 days for the perfusion loops. Statically cultured samples had complete 30 mL media changes every second day until day 9, then every day until day 14, and then were moved into 70 mL of media which was changed every second day. Scaffolds were sacrificed on day 20 or day 24.

### Histological Sectioning

At the end of the cell culture period, scaffolds were removed from the bioreactor chambers and gently washed with PBS before being placed into a custom‐designed cutting guide. A series of slots were included in the guide that allowed a microtome feather blade to precisely section the scaffold into 8 individual 3 mm thick pieces. The cutting guide was 3D printed (Makerbot Replicator 2X) from acrylonitrile butadiene styrene (ABS) thermoplastic and sterilized using 70% ethanol solution. Result slices are shown in Figure S26, Supporting Information.

### Metabolic Assays

Alamar blue cell viability reagent (Invitrogen, Massachusetts, United States) was used to measure the activity of living cells cultured within the scaffolds. Small 5 mm diameter discs were cored out using a biopsy punch from identical locations in each scaffold section. Scaffold discs were incubated in 10% (v/v) Alamar blue regent in media for 3 h at 37 °C (all Invitrogen). After incubation, the fluid was removed from the samples and read on a POLARstar Optima fluorescence plate reader (BMG LabTech, Ortenberg, Germany) at an excitation wavelength of 550 nm and emission of 590 nm. Background fluorescence samples were included in each plate, taken from scaffolds that had not been seeded with cells but had otherwise undergone identical processing, precondition, and media incubation methods.

### Immunofluorescent Microscopy

Cell viability was assessed by incubating scaffold slices for 25 min in 2 µm calcein AM and 4 µm EthD‐1 in PBS at room temperature. After incubation, samples were gently washed with PBS and immediately imaged to assess cell viability. Cell morphology was assessed by staining actin fibers and cell nuclei. Scaffold slices were gently washed with PBS three times before immersing in 4% paraformaldehyde (PFA) solution for 10 min at room temperature. After removal of PFA, scaffold slices were washed three times with PBS and then permeabilized in 0.2% (v/v) Triton X‐100 in PBS for 4 min at room temperature. Samples were then washed three times with PBS before blocking in 1% (w/v) bovine serum albumin/PBS for 20 min at room temperature, then stained in a solution of 0.165 mm Alexa Fluor 488 Phalloidin in PBS for 20 min at room temperature, protected from light. After Phalloidin staining, samples were washed 3 times with PBS, then stained in 300 nm 4',6‐Diamidino‐2‐Phenylindole (DAPI) (all Invitrogen, Mulgrave, Australia) in PBS for 3 min before gently washing three times with PBS. Samples were stored in PBS protected from light. Detailed cell morphology and micro (scaffold pore scale) distributions were visualized with a Leica SP5 Confocal Laser Scanning Microscope (Leica, Wetzlar, Germany). Macro (full section thickness) cell viability distribution was imaged on a Zeiss Axio Imager M2 Fluorescent Microscope (Carl Zeiss AG, Oberkochen, Germany) using tile scanning on a motorized stage to resolve the entire slice at high magnification.

### Electron Microscopy

Detailed morphology of the cells and scaffold substrate were visualized using a Tescan Mira3 (Tescan, Brno, Czech Republic) scanning electron microscope. The result slices were taken from the cutting guide and a 5 mm diameter biopsy punch was used to core out sample discs from specific locations throughout the scaffold. Samples were fixed in 3% glutaraldehyde and then soaked three times with 0.1 m cacodylate buffer for 10 min per soak. The samples were then immersed in 1% osmium tetroxide in cacodylate buffer for 1 h, before being soaked for 10 min with ultrapure water twice, followed by sequential ethanol dehydration. Samples were immersed in 50%, 70%, and 90% ethanol twice each for 10 min, then twice with 100% ethanol for 15 min each immersion. The final drying step consisted of twice immersing the samples in hexamethyldisilazane for 30 min, then air drying on filter paper for 12 h before storing in low humidity conditions (all Thermo‐Fisher, Waltham, USA). Dry samples were coated with gold using a Leica EM SCD005 sputter coater before imaging.

## Conflict of Interest

The authors declare no conflict of interest.

## Supporting information

Supporting Information

## Data Availability

The data that support the findings of this study are available from the corresponding author upon reasonable request.
