# Supplementary material for: Personalized Volumetric Tissue Generation by Enhancing Multiscale Mass Transport through 3D Printed Scaffolds in Perfused Bioreactors
Source: Adv Healthc Mater. 2022 Jul 8;11(24):2200454. doi: 10.1002/adhm.202200454 (PMC11468985; doi:10.1002/adhm.202200454)
Supplement: Supplementary file 1 — Supporting Information [file ADHM-11-2200454-s001.pdf]

ADVANCED
HEALTHCARE
MATERIALS

Supporting Information

for *Adv. Healthcare Mater.*, DOI 10.1002/adhm.202200454

Personalized Volumetric Tissue Generation by Enhancing Multiscale Mass Transport through 3D Printed Scaffolds in Perfused Bioreactors

*David P Forrestal, Mark C Allenby, Benjamin Simpson, Travis J Klein and Maria A Woodruff**

Supporting Information

Title Personalised volumetric tissue generation by enhancing multiscale mass transport through 3D printed scaffolds in perfused bioreactors

David P Forrestal,[1,2,3] Mark C Allenby,[1,4] Benjamin Simpson,[5] Travis J Klein,[1] Maria A Woodruff [1]*

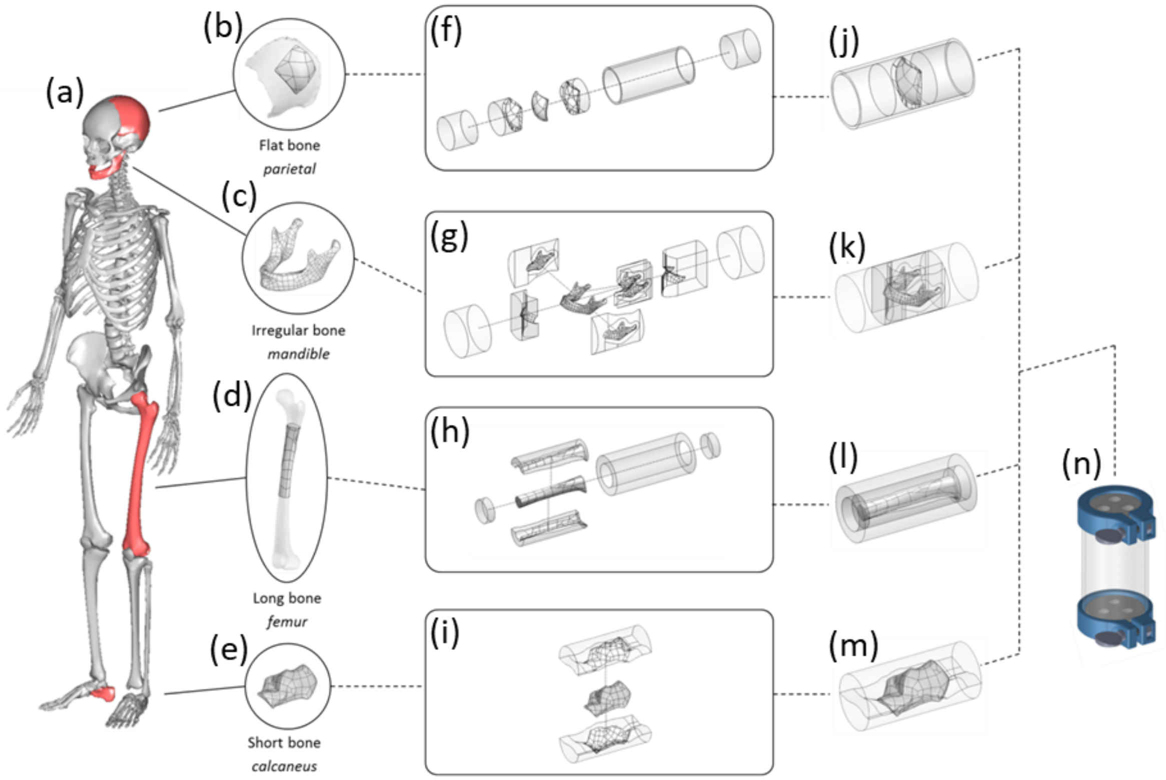

Figure S1: (a) Bone implants can be required in a variety of shapes and sizes due to trauma, congenital abnormalities, and surgical interventions. These implant scaffolds can be for defects in (b) flat bones such as the parietal, (c) irregular bones such as the mandible, (d) long bones such as the femur, and (e) short thick bones such as the calcaneus. (f-i) Close-fitting inserts can be provided around these implant shapes which disassemble and reassemble if separated along planes of surface angle transition with respect to assembly direction. (j-m) These inserts can be useful in providing methods of customising the delivery of culture media flow to the scaffolds within the chamber so that a standard bioreactor shell (n) can incorporate a range of implant sizes and shapes.

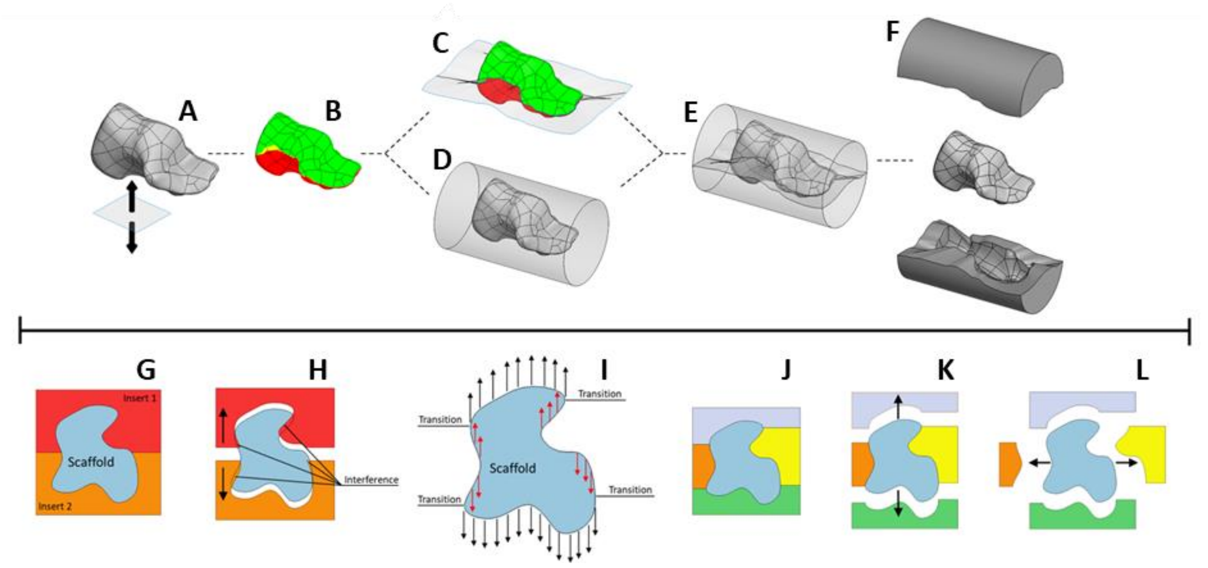

Figure S2. (A) Creation of splitting inserts requires determining assembly direction, (B) marking the scaffold where the surface angle is parallel to the assembly direction (transition from green to red surface colour), (C-F) splitting the insert at this location to result in insert components that completely enclose and conform to the surface of the scaffold while also allowing assembly and disassembly. (G-L) Complex scaffolds may require additional inserts which assembly to the scaffold from different directions.

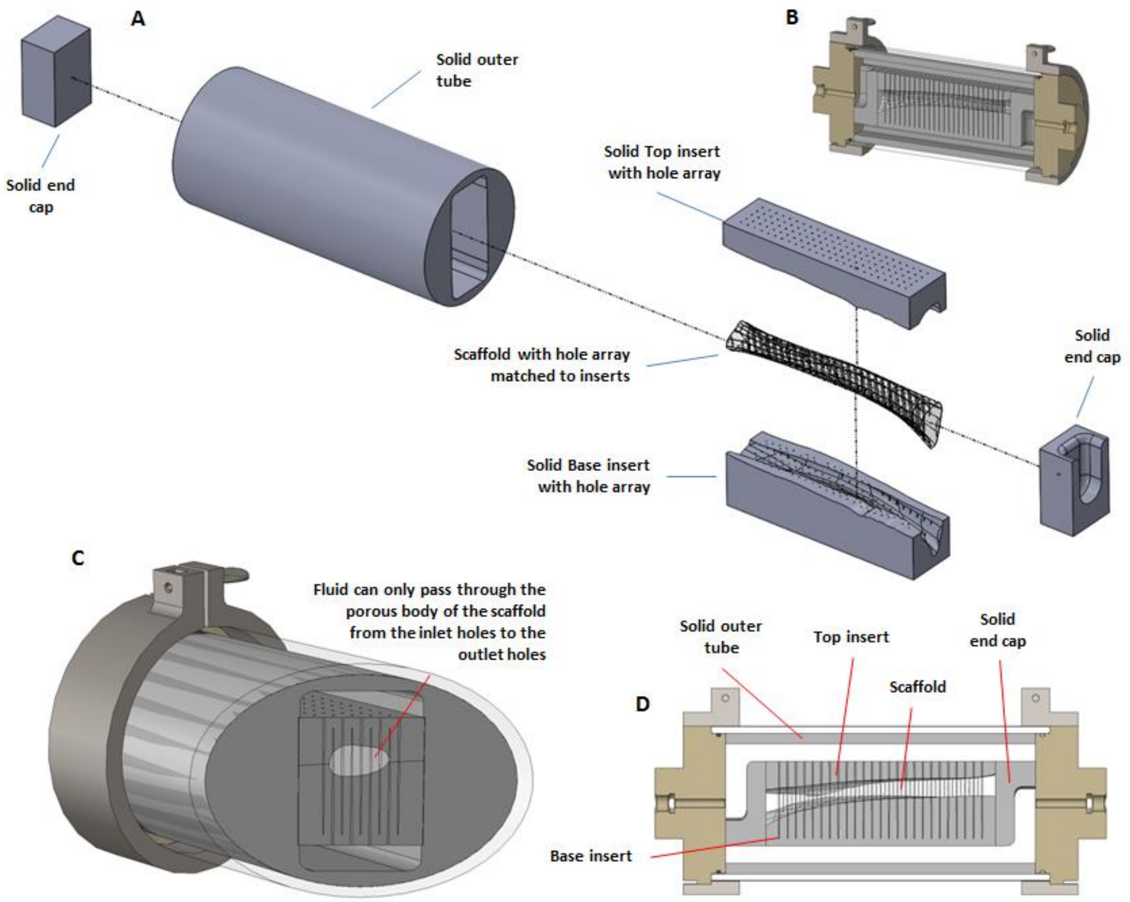

Figure S3. Exploded view of the *injections* concept applied to a femur scaffold (A). Isometric view (B), cross section view (C) and cross section view (D).

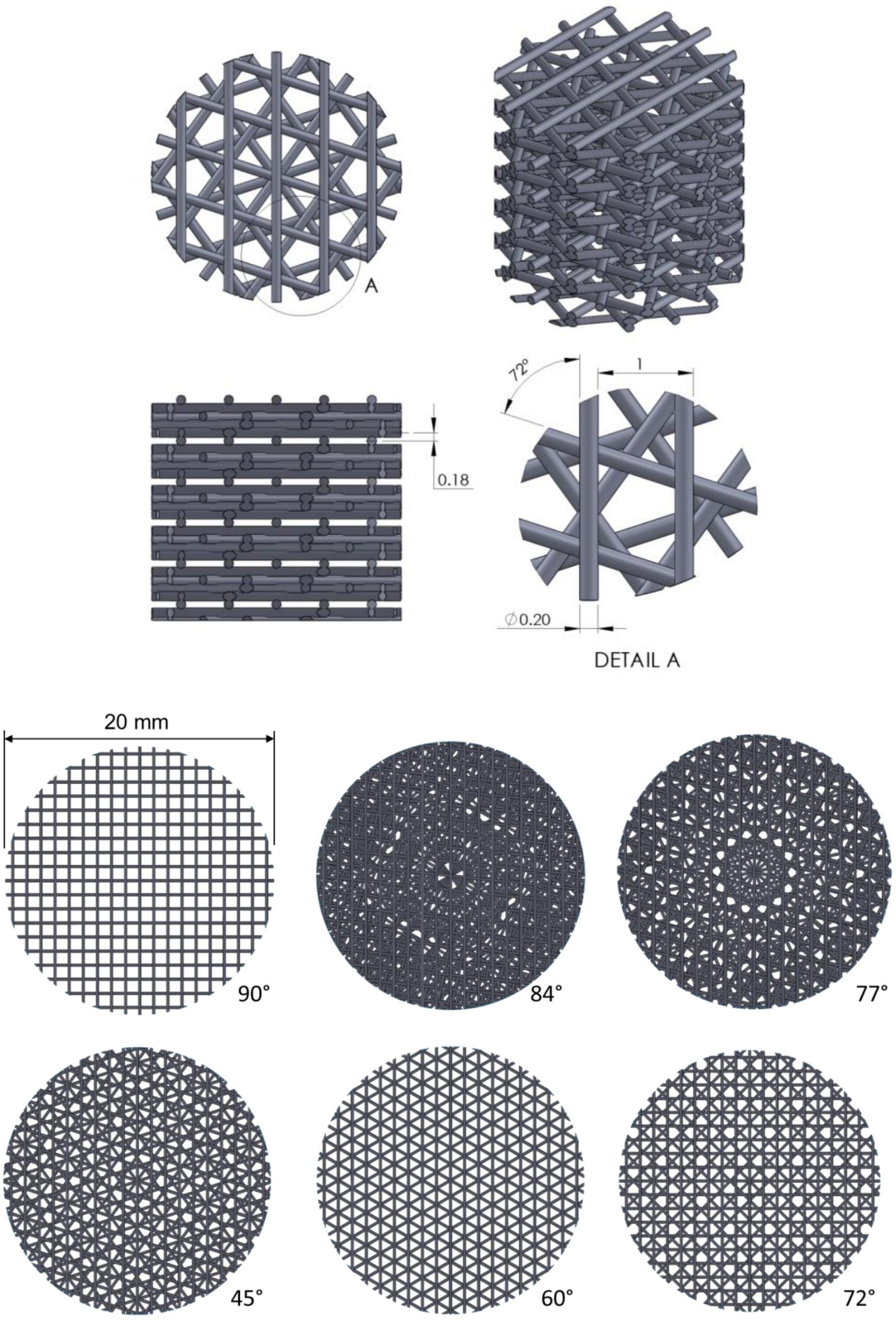

Figure S4. (top) Computer generated model showing a parametric scaffold with 72° layer offset angle and porous structure. (bottom) Parametric scaffold layer offset angle variation.

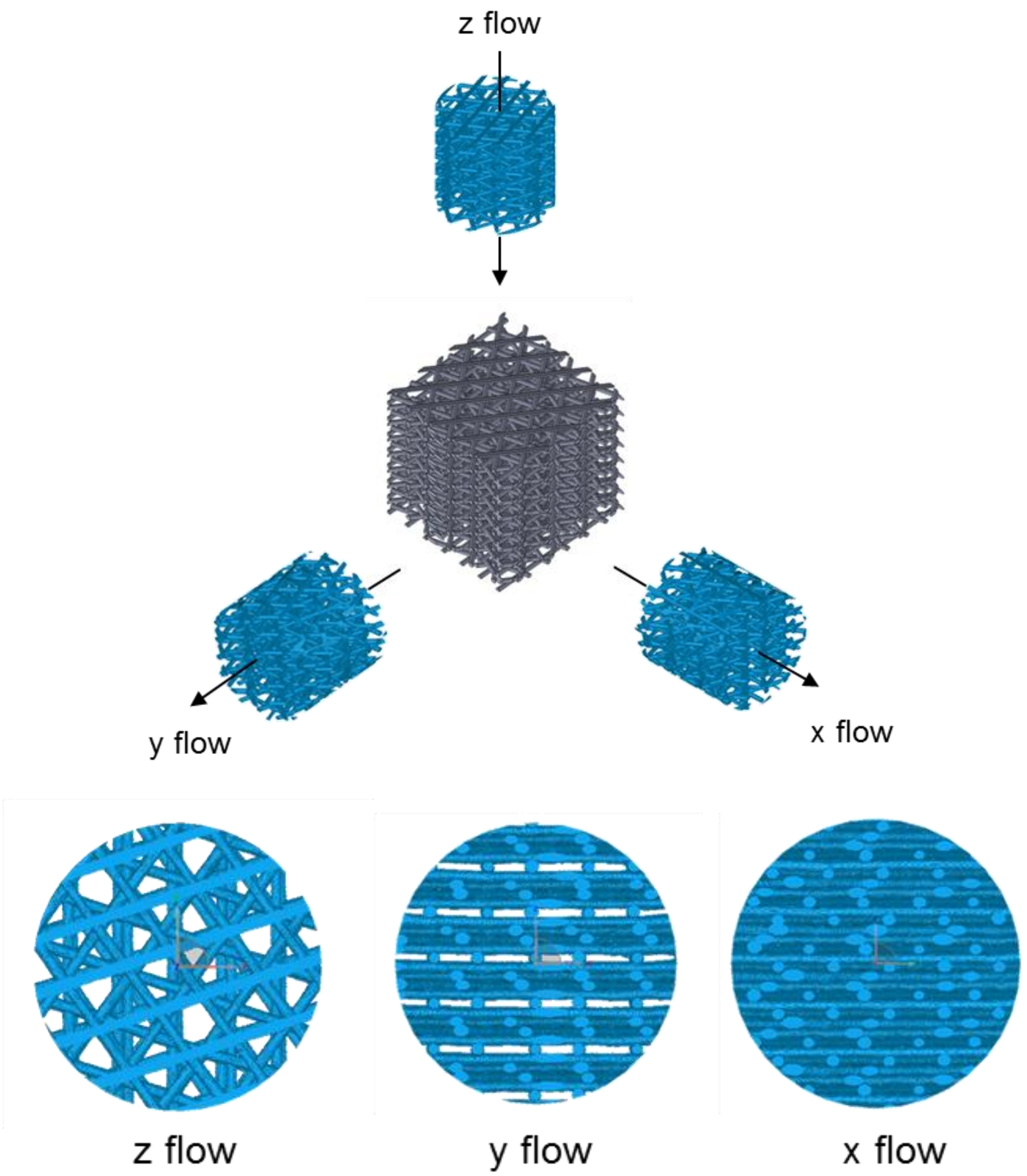

Figure S5. 3D computer generated models showing the three CFD simulation scaffold samples and the direction of simulated fluid flow through each sample. All modes were extracted from the same ROI that was created from a µCT scan of a manufactured scaffold sample.

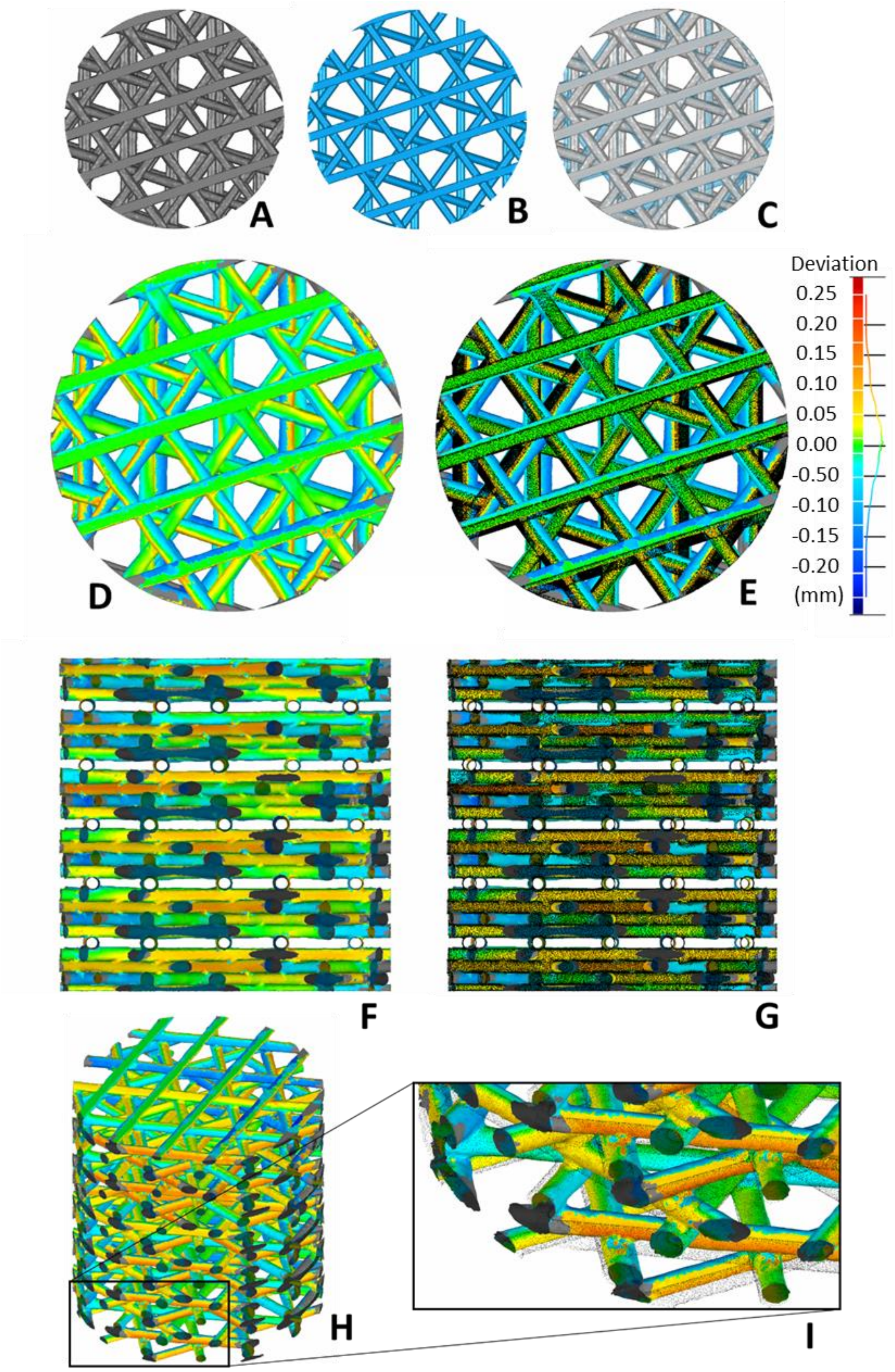

Figure S6. Surface deviation analysis between the µCT (A) and CAD (B) derived scaffold geometry used in the CFD simulations. The µCT geometry shown in grey is overlayed with the CAD derived geometry in transparent blue (C). The deviation analysis shows the µCT geometry coloured by distance in mm from the nearest point on the CAD surface mesh (D, F

and H). The CAD geometry test points are shown overlayed onto the 3D deviation plot as black dots (E, G and I). A histogram adjacent to the legend shows the distribution of the measured deviation results. Analysis performed in Geomagic Wrap (version 2015.1.1, 3D systems). Alignment of the µCT and CAD structures was performed manually.

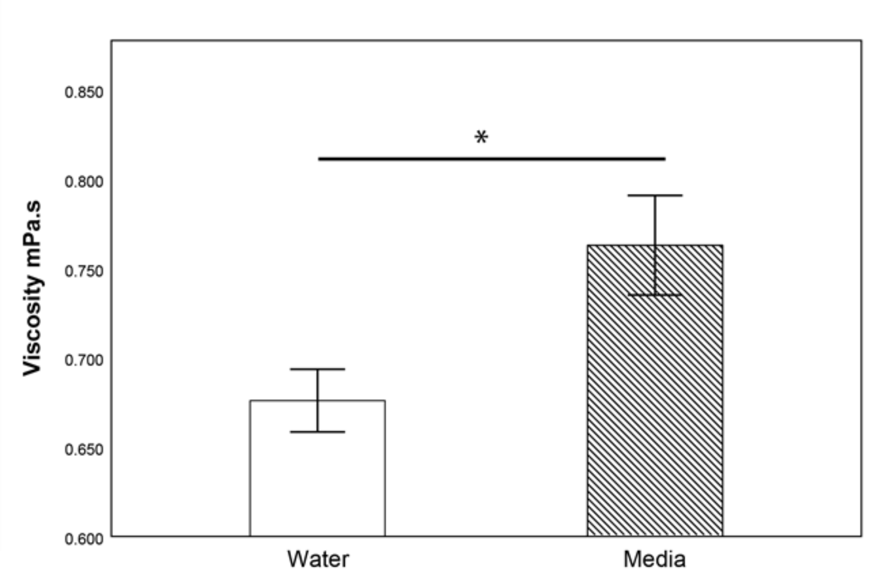

Figure S7. (top) Viscosity of culture media compared to water at 37 °C. Star indicates significant difference between groups ($P < 0.05$, $n = 112$). Error bars show standard deviation.

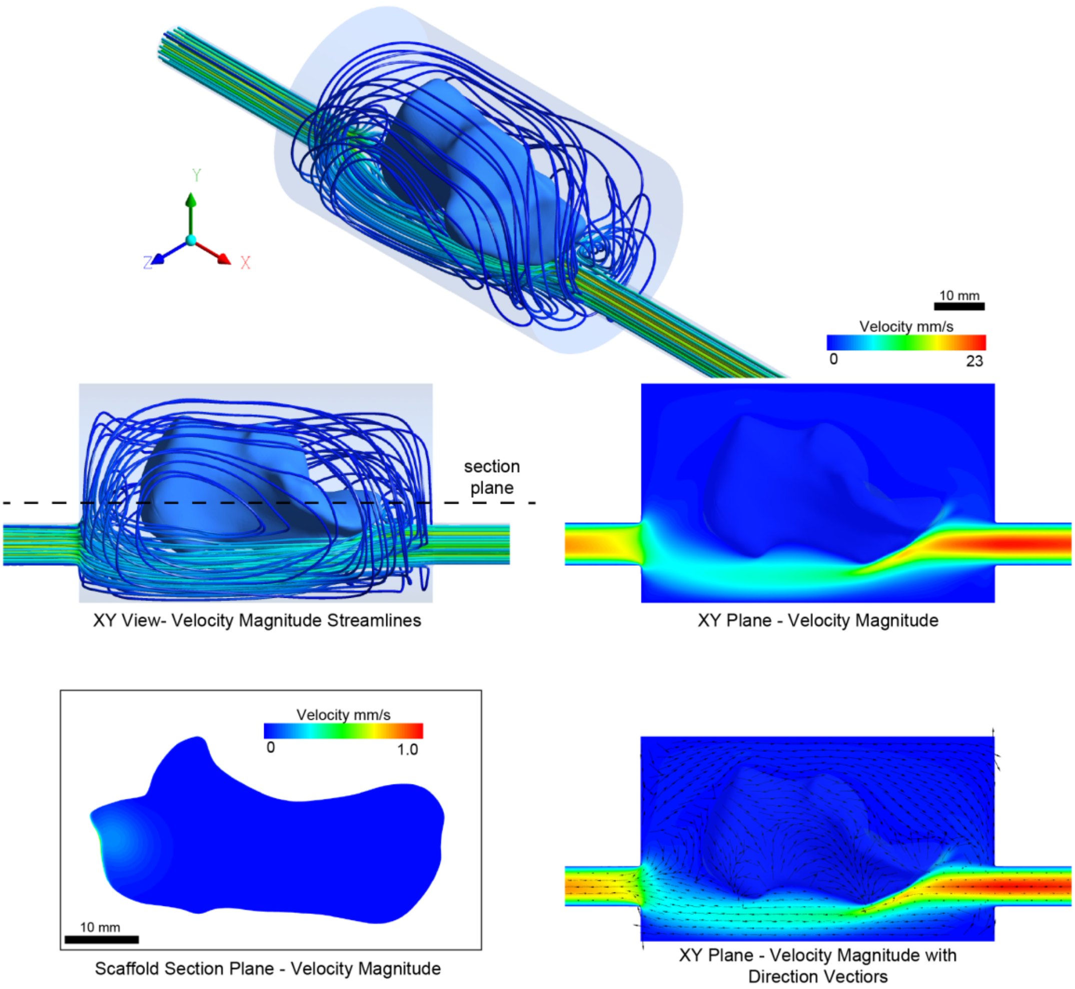

Figure S8. CFD results for the open chamber method. Isometric view of calcaneus scaffold within fluid chamber and streamlines (coloured by velocity magnitude) indicating flow paths from inlet to outlet (top). Side view of calcaneus scaffold with fluid streamlines coloured by velocity magnitude (middle left). Cross section through the XY plane showing a contour plot of velocity magnitude (middle right). Cross section through the XY plane showing a velocity magnitude contour plot and velocity direction vector arrows (bottom right). Section view showing velocity magnitude within the scaffold zone only (bottom left).

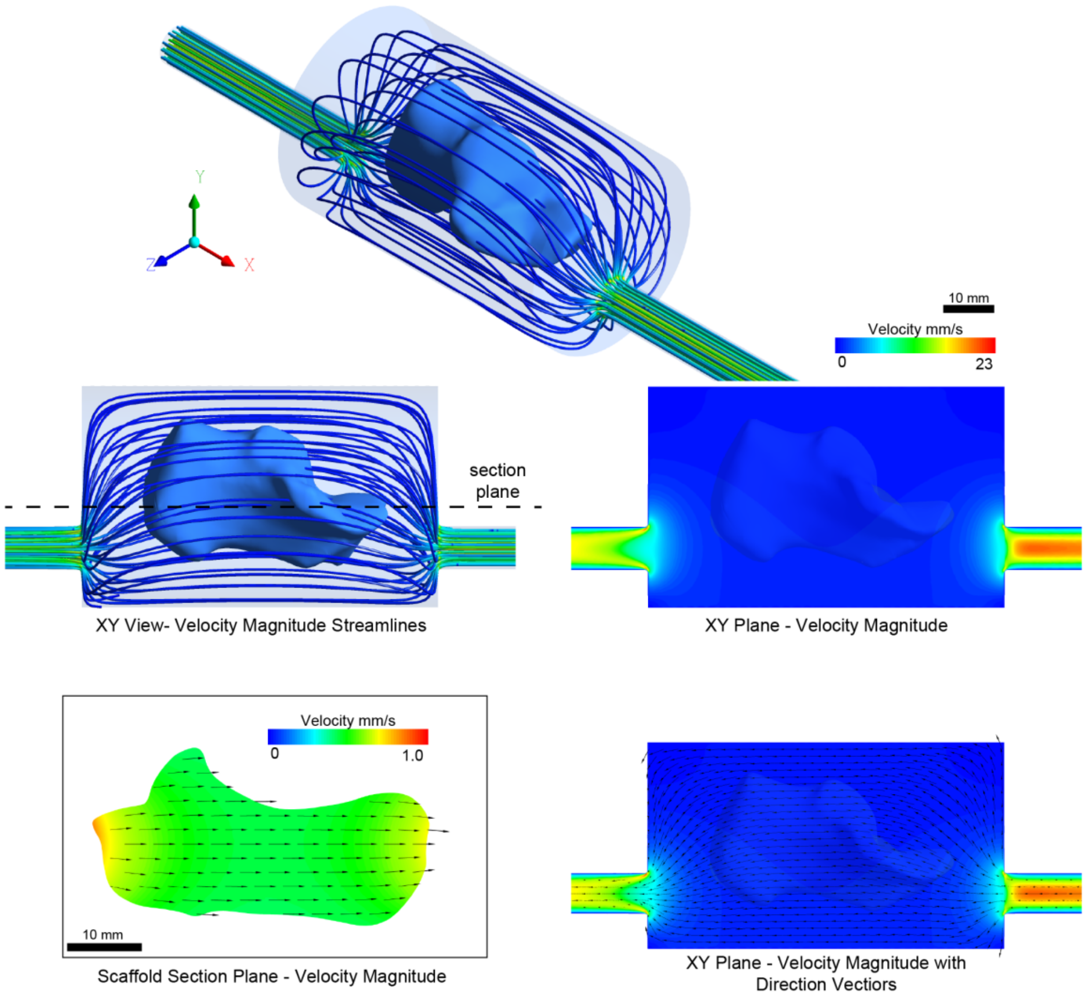

Figure S9. CFD results for the diffuser method. Isometric view of calcaneus scaffold within fluid chamber and streamlines (coloured by velocity magnitude) indicating flow paths from inlet to outlet (top). Side view of calcaneus scaffold with fluid streamlines coloured by velocity magnitude (middle left). Cross section through the XY plane showing a contour plot of velocity magnitude (middle right). Cross section through the XY plane showing a velocity magnitude contour plot and velocity direction vector arrows (bottom right). Section view showing velocity magnitude within the scaffold zone only (bottom left).

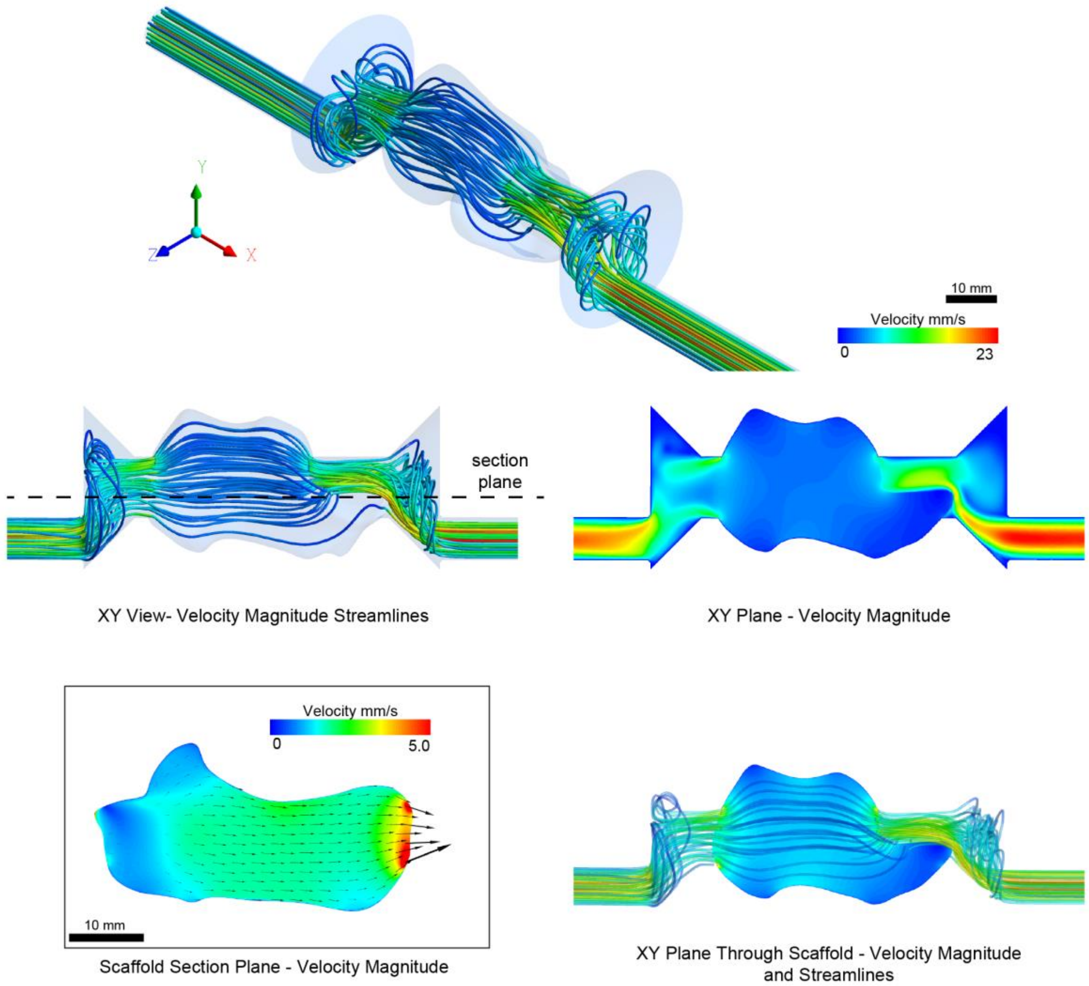

Figure S10. CFD results for the cassette method. Isometric view of calcaneus scaffold within fluid chamber and streamlines (coloured by velocity magnitude) indicating flow paths from inlet to outlet (top). Side view of calcaneus scaffold with fluid streamlines coloured by velocity magnitude (middle left). Cross section through the XY plane showing a contour plot of velocity magnitude (middle right). Cross section through the XY plane showing a velocity magnitude contour plot and velocity direction vector arrows (bottom right). Section view showing velocity magnitude within the scaffold zone only (bottom left).

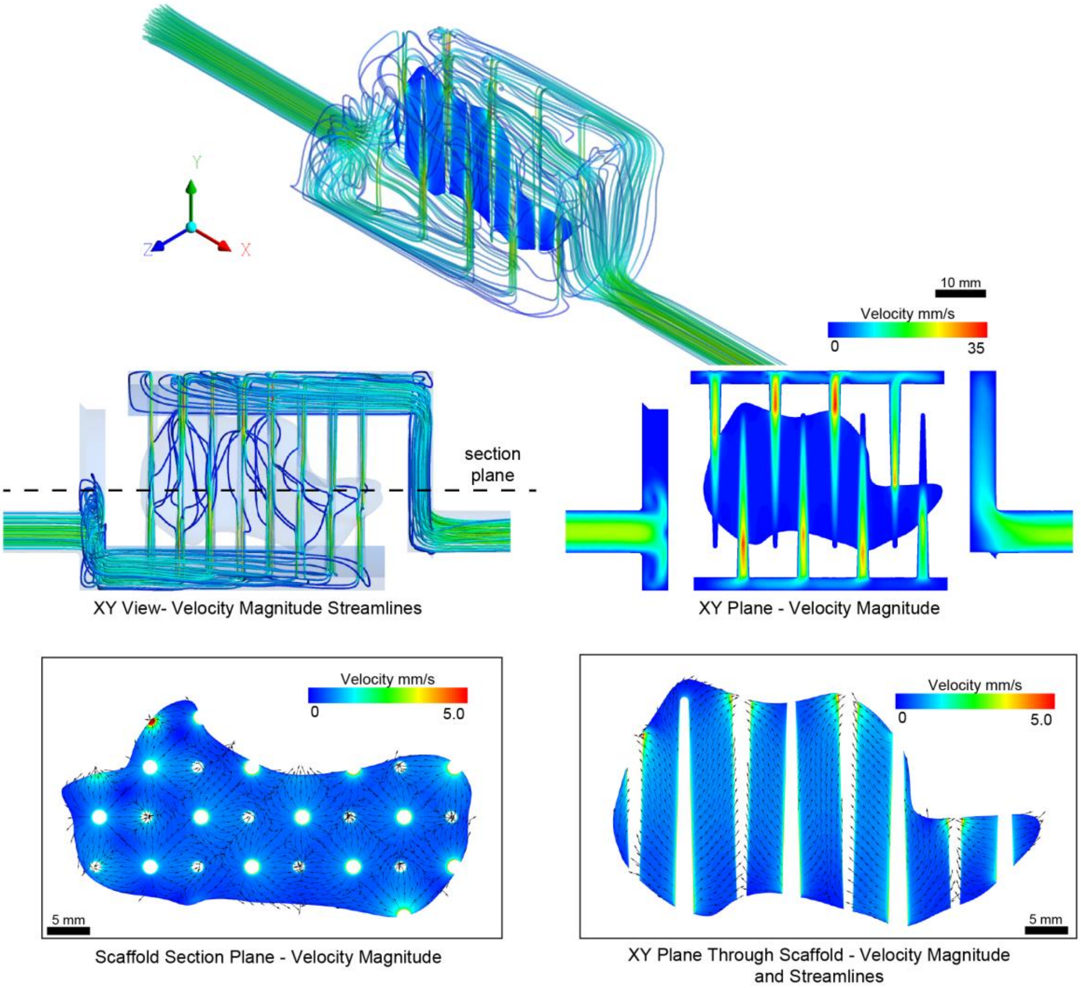

Figure S11. CFD results for the injections method. Isometric view of fluid zone with central plane XY plane through scaffold coloured by velocity magnitude, streamlines (coloured by velocity magnitude) indicate flow paths from inlet to outlet (top). Side view of calcaneus scaffold with fluid streamlines coloured by velocity magnitude (middle left). Cross section through the XY plane showing a contour plot of velocity magnitude (middle right). Cross section through the scaffold XY plane showing a velocity magnitude contour plot and velocity direction vector arrows (bottom right). Section view showing velocity magnitude and velocity direction vectors within the scaffold zone only (bottom left).

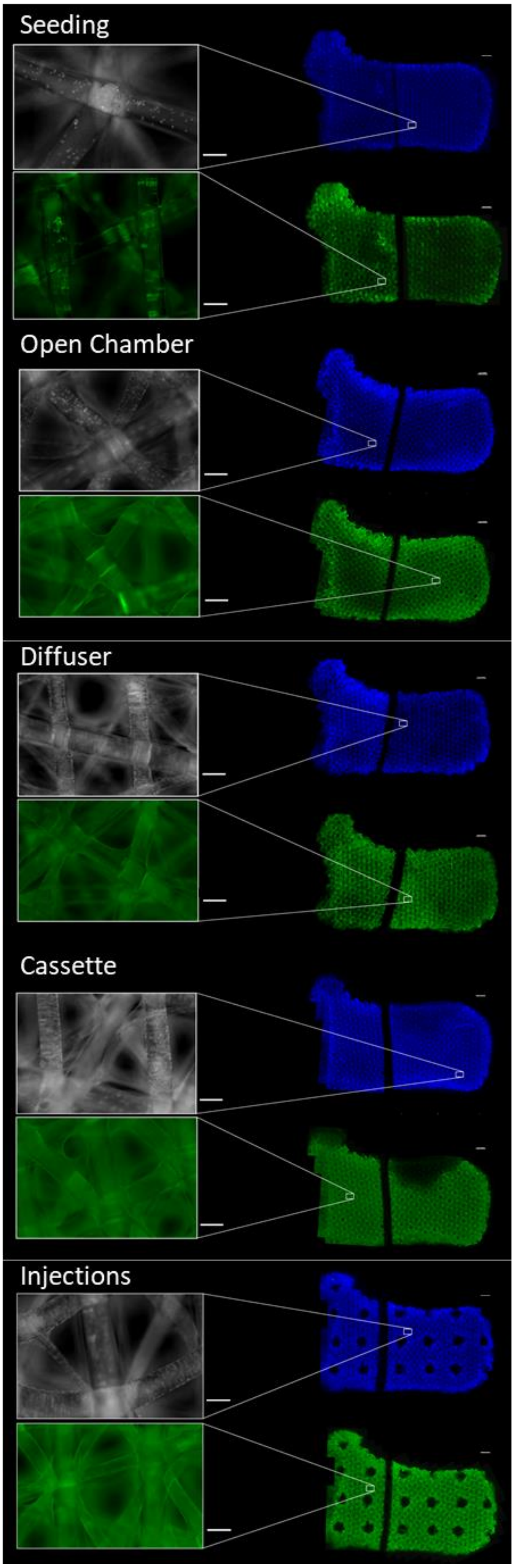

Figure S12. Fluorescence micorscopy images of cell cytoskeleton and nuclei throughout whole-bioreactor cross sections at day 7 alongside magnified microscopy images at the core of the scaffold. Microscopy samples were stained by phalloidin-conjugated AlexaFluor 488 and 4′,6-diamidino-2-phenylindole. The bioreactor scaffold cross-sections had to be cut in half to fit within an imaging plate. ROI scale bars are 200 µm (left insert images) and the whole-bioreactor cross-sections scale bars are 2000 µm. These bioreactor cross-sections were not sectioned from the exact centre of the scaffold, which accounts for their varied shape versus Figures 5 and 6.

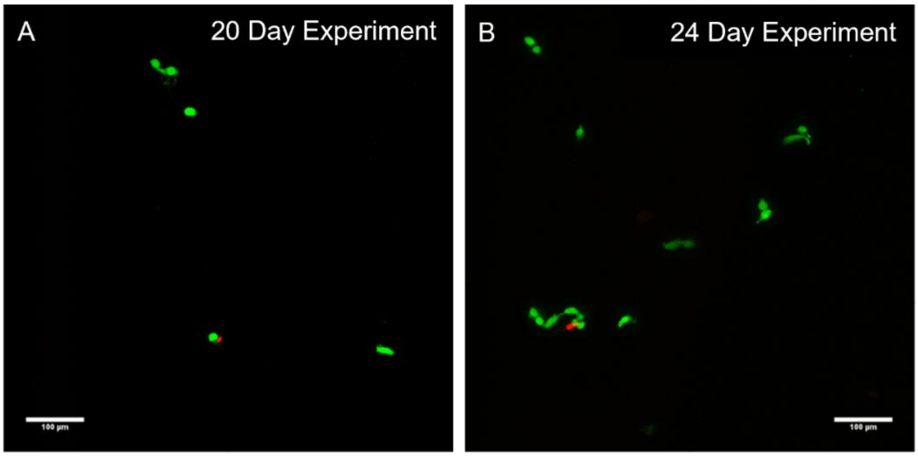

Figure S13. CLSM of seeding sample scaffolds cultured statically for 24 hrs and stained for live (green, calcein AM) and dead (red, EthD-1) cells. (A) Seeding scaffold for 20 day culture experiment. (B) Seeding scaffold for 24 day culture experiment. All scale bars 100 µm.

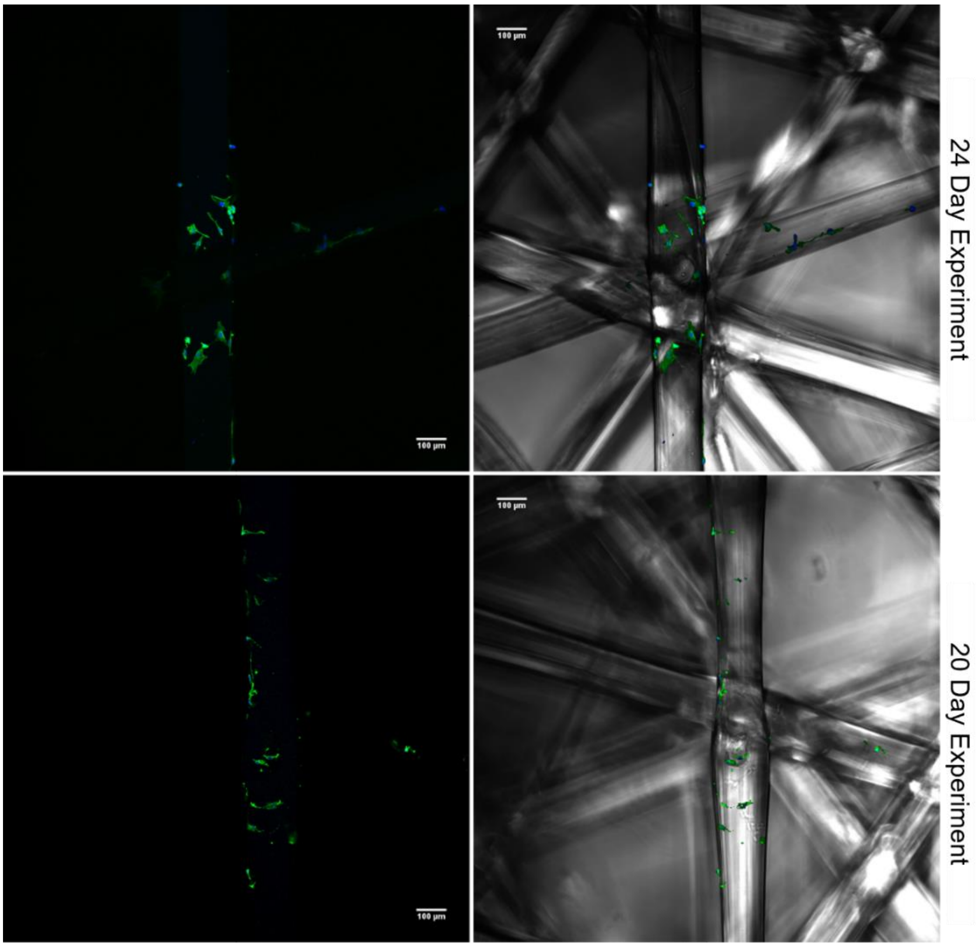

Figure S14. CLSM images of seeded sample scaffolds cultured statically for 24 hrs and stained for actin (green, Phalloidin) and nucleus (blue, DAPI) cells. Maximum projection images on left and bright field images overlayed on the right. Images are from the central zone of the scaffold for both groups. All scale bars 100 µm.

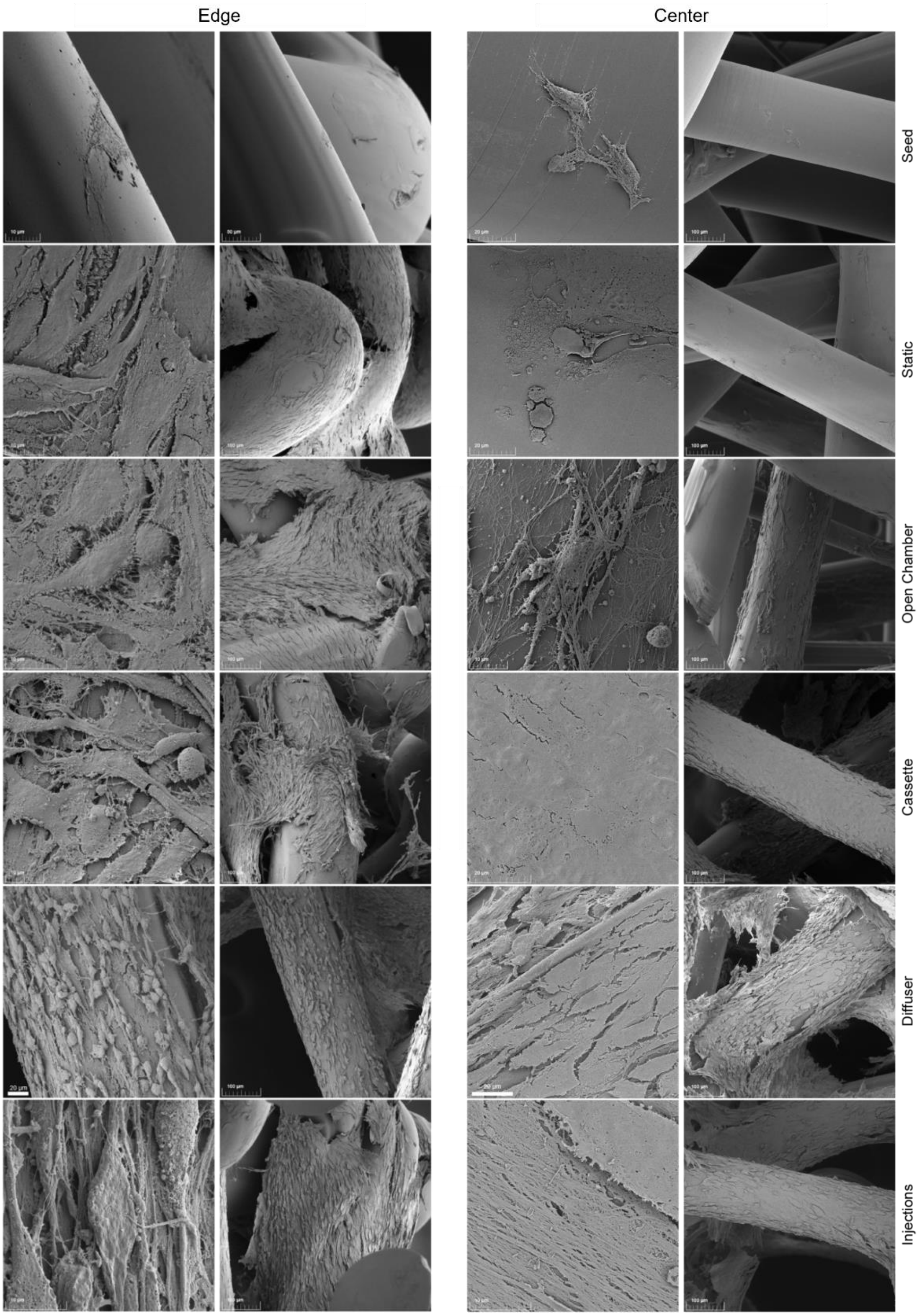

Figure S15. SEM images for day 20 scaffolds. Regions close to the edge are within 2000 µm of the scaffold surface (left panel), and central regions deeper than 2000 µm from the scaffold surface (right). Low and high magnification images all images have the same central position.

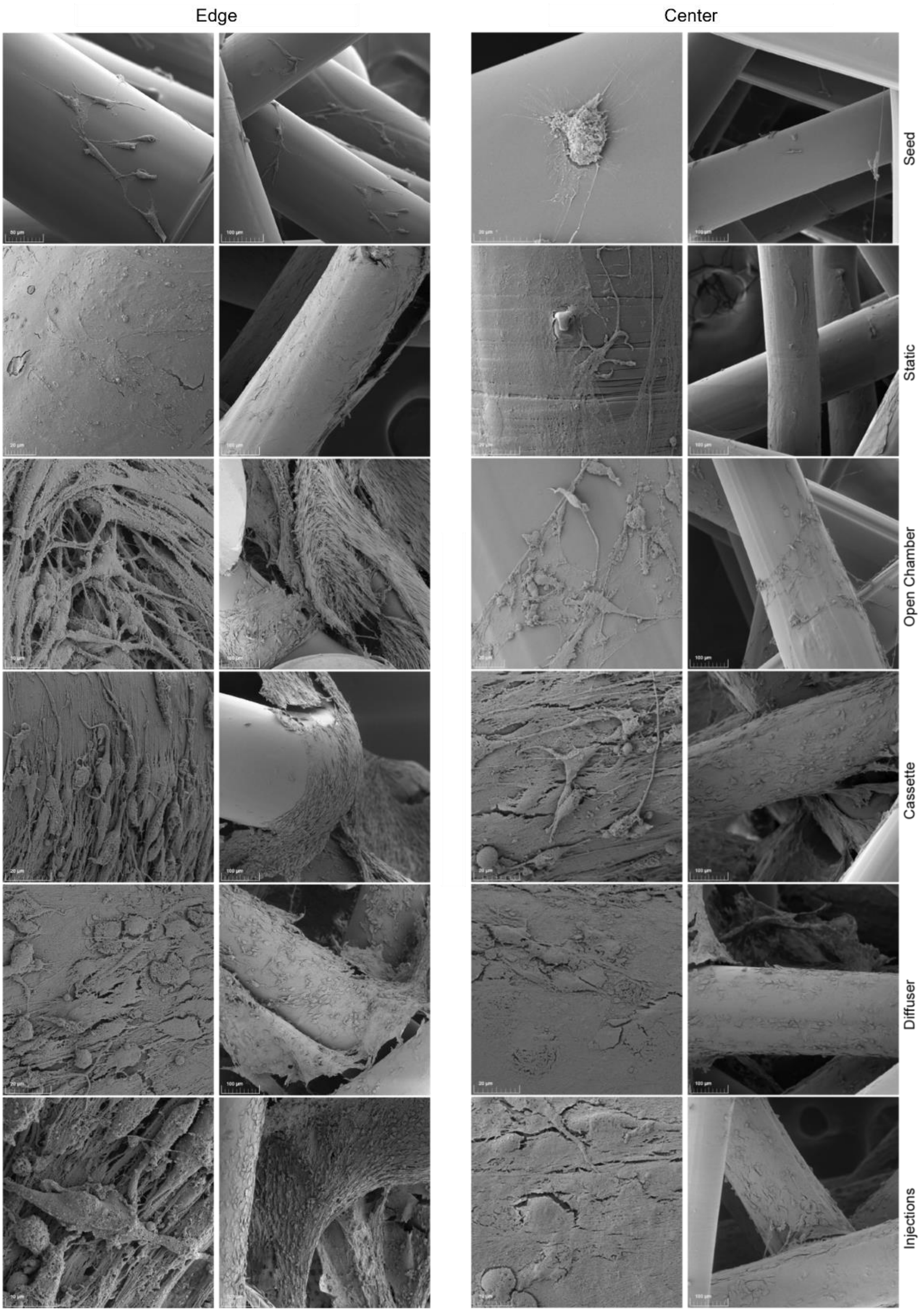

Figure S16. SEM images for day 24 scaffolds. Regions close to the edge are within 2000 µm of the scaffold surface (left panel), and central regions deeper than 2000 µm from the scaffold surface (right). Low and high magnification images all images have the same central position.

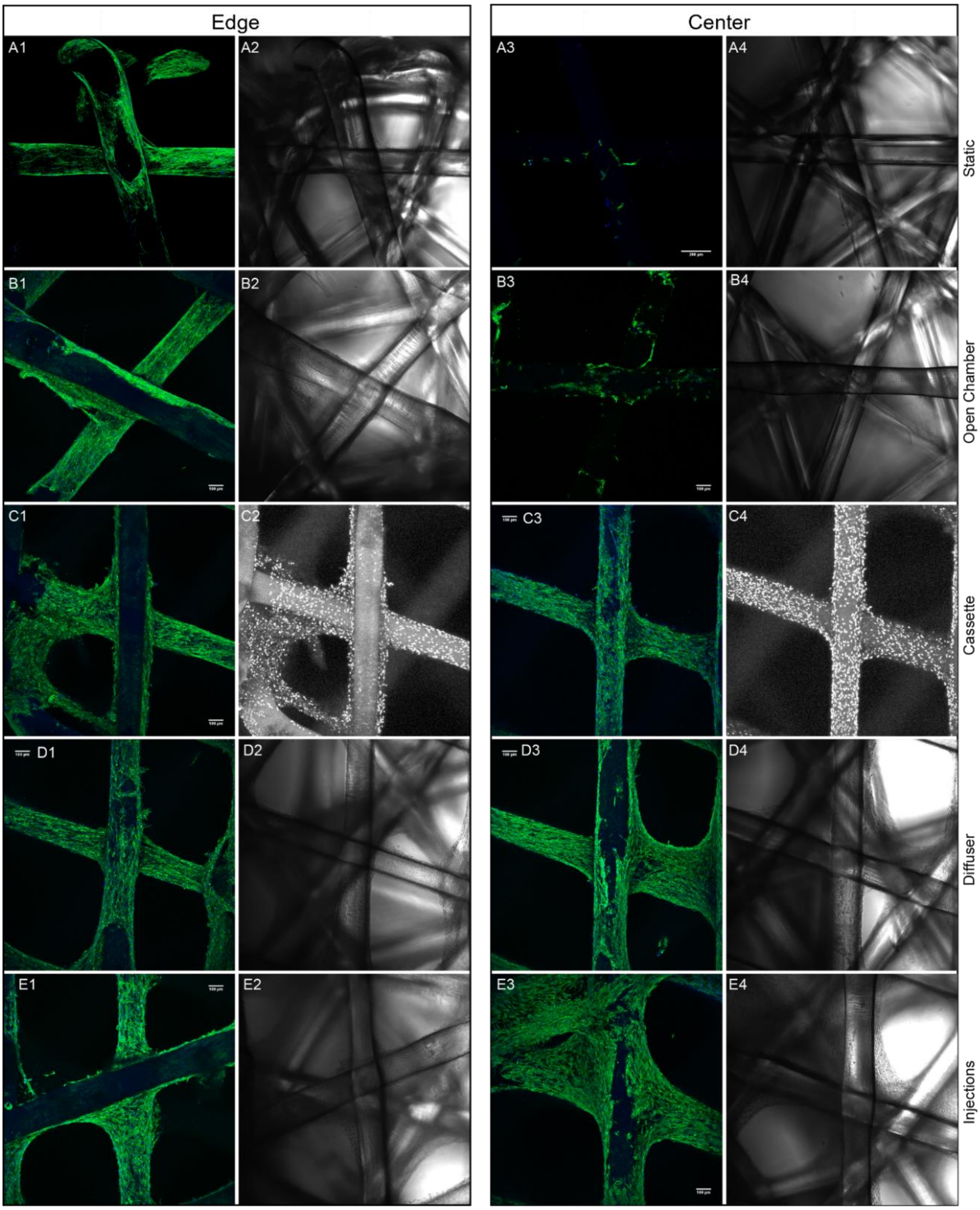

Figure S17. CLSM images of day 20 scaffolds stained for actin (green, Phalloidin) and nuclei (blue, DAPI). Bright field images of the identical region are provided to the right of each fluorescent image. Figure C2 and C4 are grayscale LUTs (look up table) of the DAPI channels from adjacent images, with the intensity distribution cropped to 0 – 50 of the 255 (8-bit) intensity values (performed in ImageJ). This shows cell nuclei and background fluorescence on the scaffold fibres. All scale bars 100 µm.

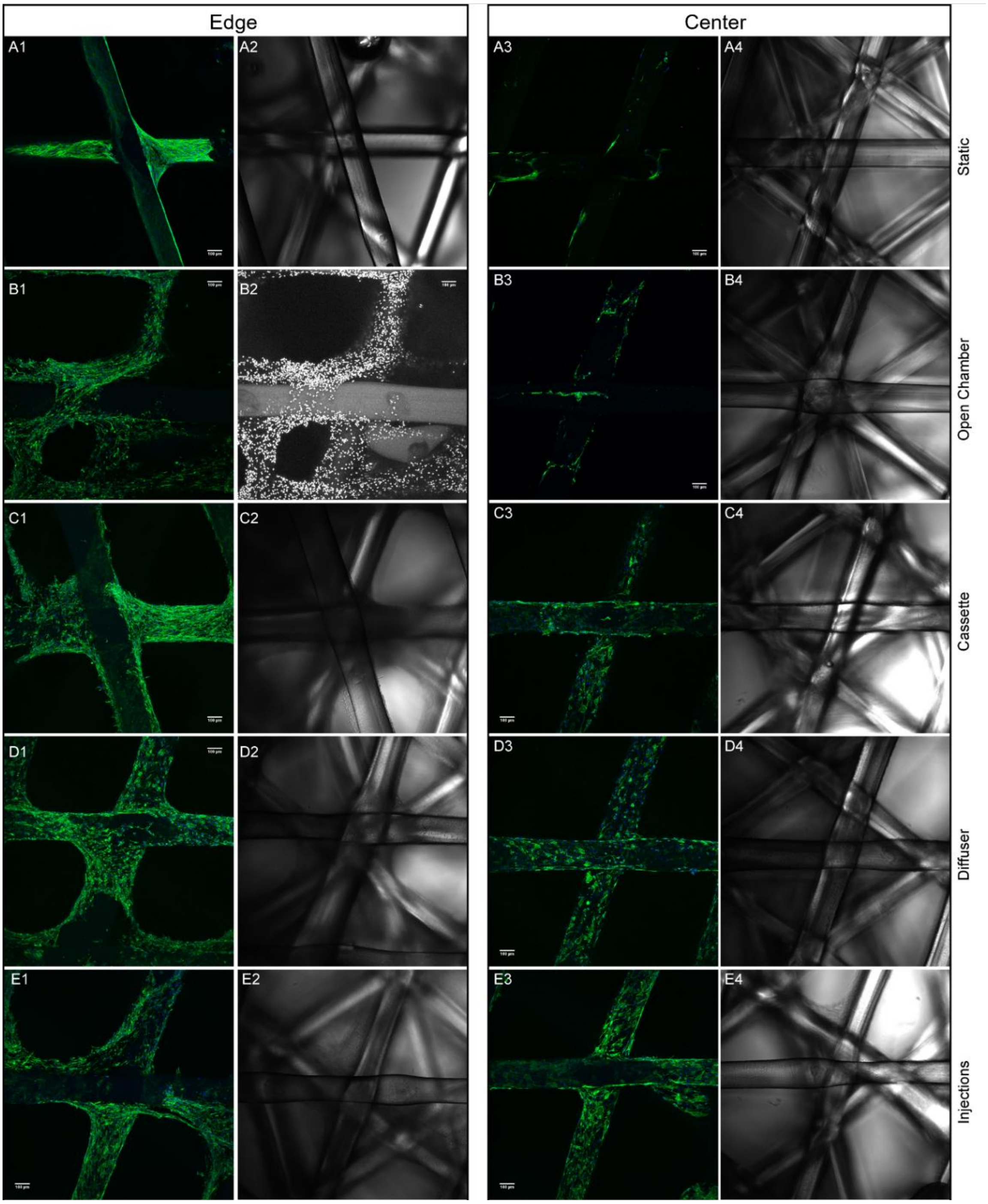

Figure S18. CLSM images of day 24 scaffolds stained for actin (green, Phalloidin) and nucleus (blue, DAPI). Bright field images of the identical ROI are provided to the right of each fluorescent image. Figure B2 is a grayscale LUT (look up table) of the DAPI channel from the B1 image, with the intensity distribution cropped to 0 – 50 of the 255 (8-bit) intensity values (performed in ImageJ). This shows cell nuclei and background fluorescence on the scaffold fibres. All scale bars 100 µm.

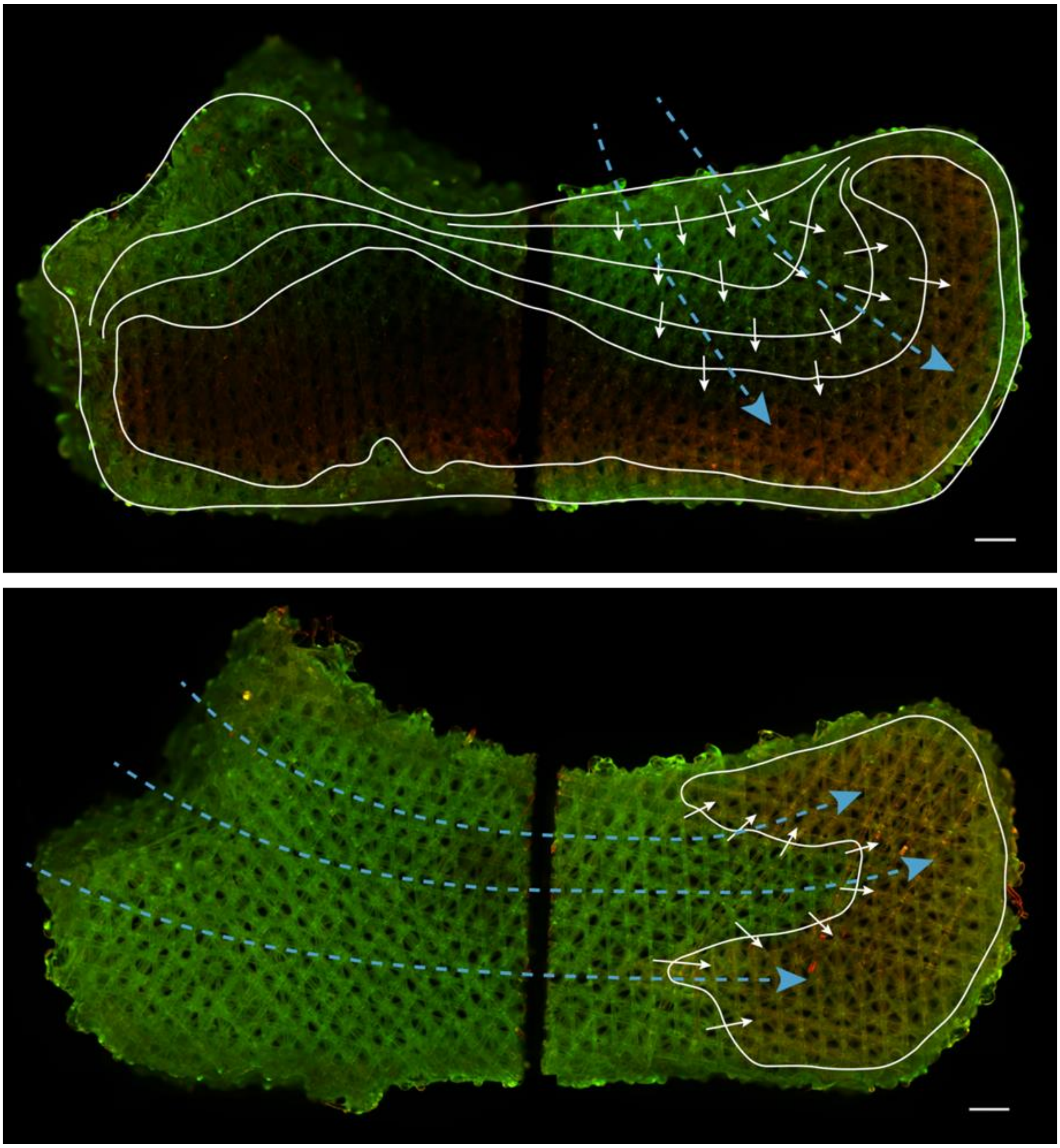

Figure S19. (top image) The day 20 ***open chamber*** scaffold live/dead fluorescent stain result (Calcein AM in green and ethidium homodimer-1 in red) with isolines drawn at boundaries of similar viability signal intensity. White arrows indicate the suspected direction of culture media dissolved species gradients. Blue arrows indicate suspected flow direction. (bottom images) The day 20 ***diffuser*** scaffold live/dead fluorescent result with isolines drawn at constant viability signal. White arrows indicate the direction of culture media dissolved species gradients. Blue arrows indicate suspected flow direction. Scale bars are 2000 μm.

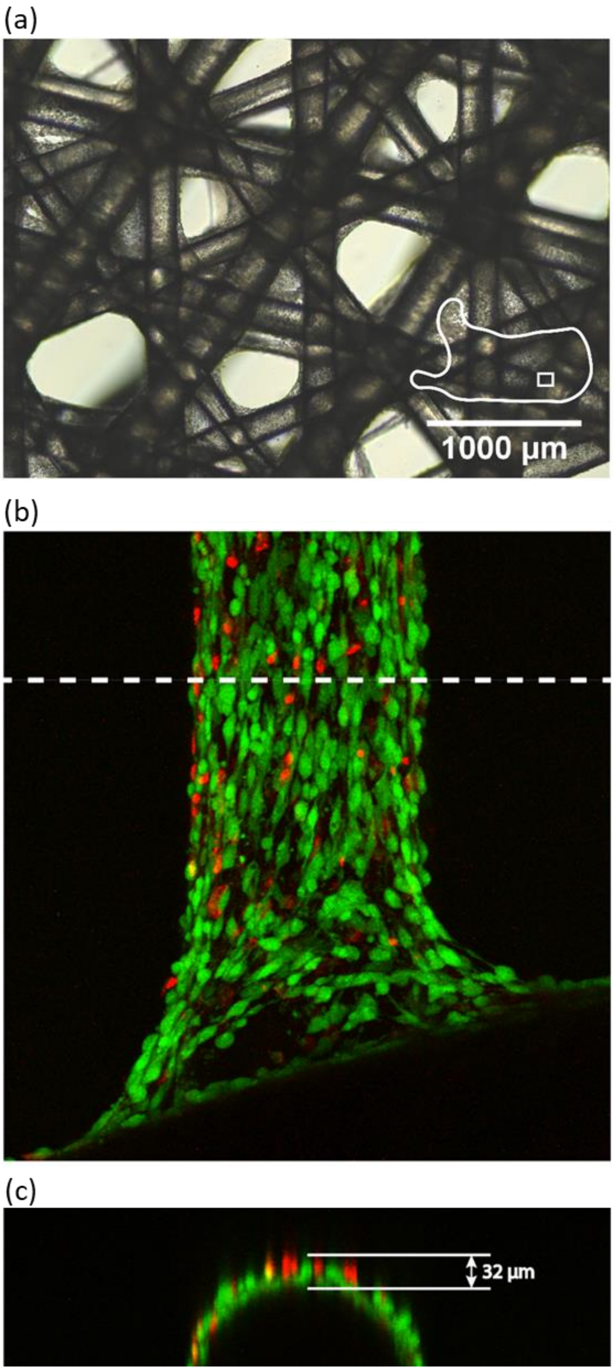

Figure S20. (A) Bright field image of the day 24 *cassette* scaffold, with bottom-right inset illustration indicating the image's location amongst the greater scaffold section geometry and scale bar. Extensive neotissue growth is apparent, with webs of cells extending between fibres and across pores. (B) CLSM maximum projection image of the day 20 diffuser scaffold live/dead result showing complete coverage of the fibre surface and the formation of a small web of cells at the fibre intersection overlaid with a dashed line depicting the cross-section image in (C). A maximum projection (50 µm depth) in the direction along the fibre axis (vertical direction on this page), centred on the dashed line in the upper image. The thickness of the confluent layer of cells is approximately 32 µm.

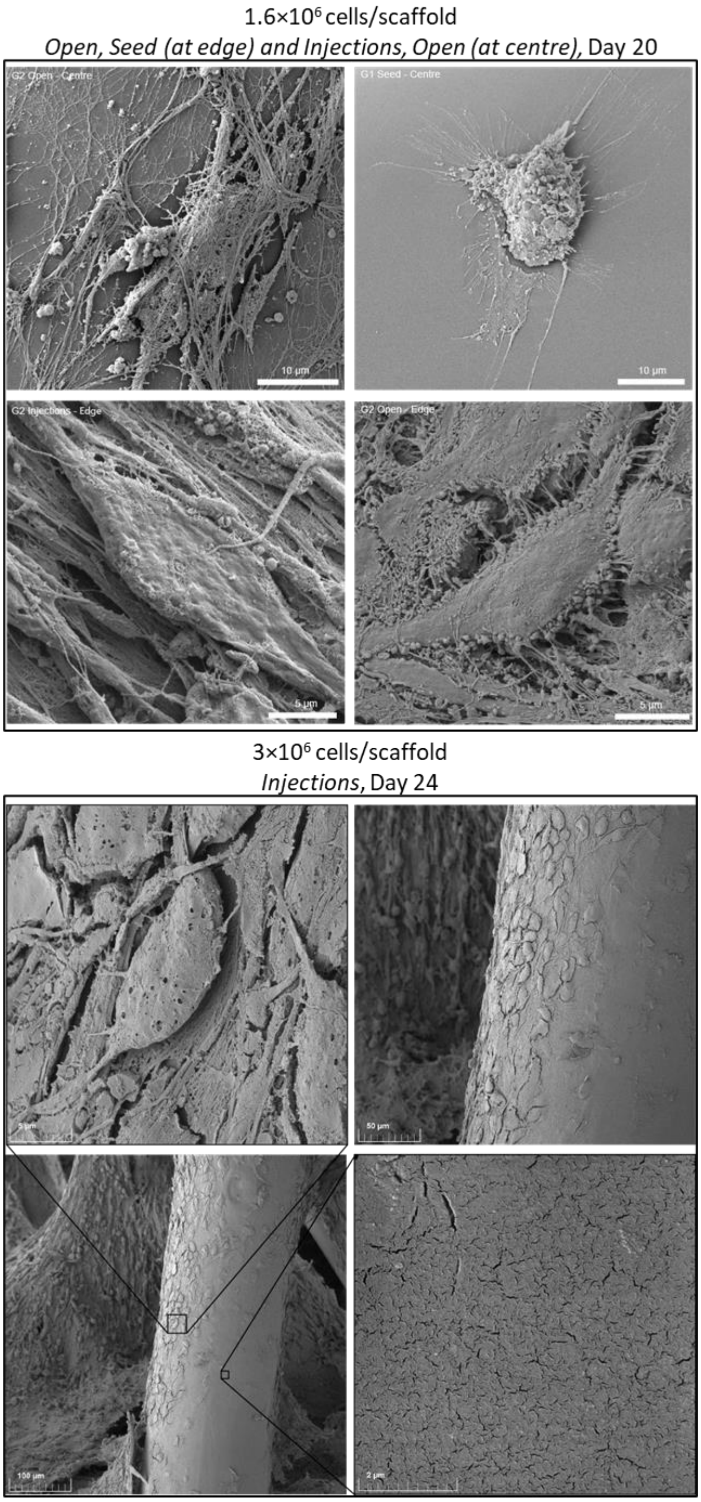

Figure S21. SEM images comparing cell morphology on fibres for (top) bioreactors seeded with 1.6×10^6 cells per scaffold and cultured for 20 days. The top row shows few cells attached on fibres at the centre of *seeding* and *open* bioreactors as compared to a confluent number of cells attached at the edge of *injections* or *open* scaffolds. (bottom) Images of *injection* bioreactor fibres at the centre of a 24 day culture with 3×10^6 cells per scaffold seeded.

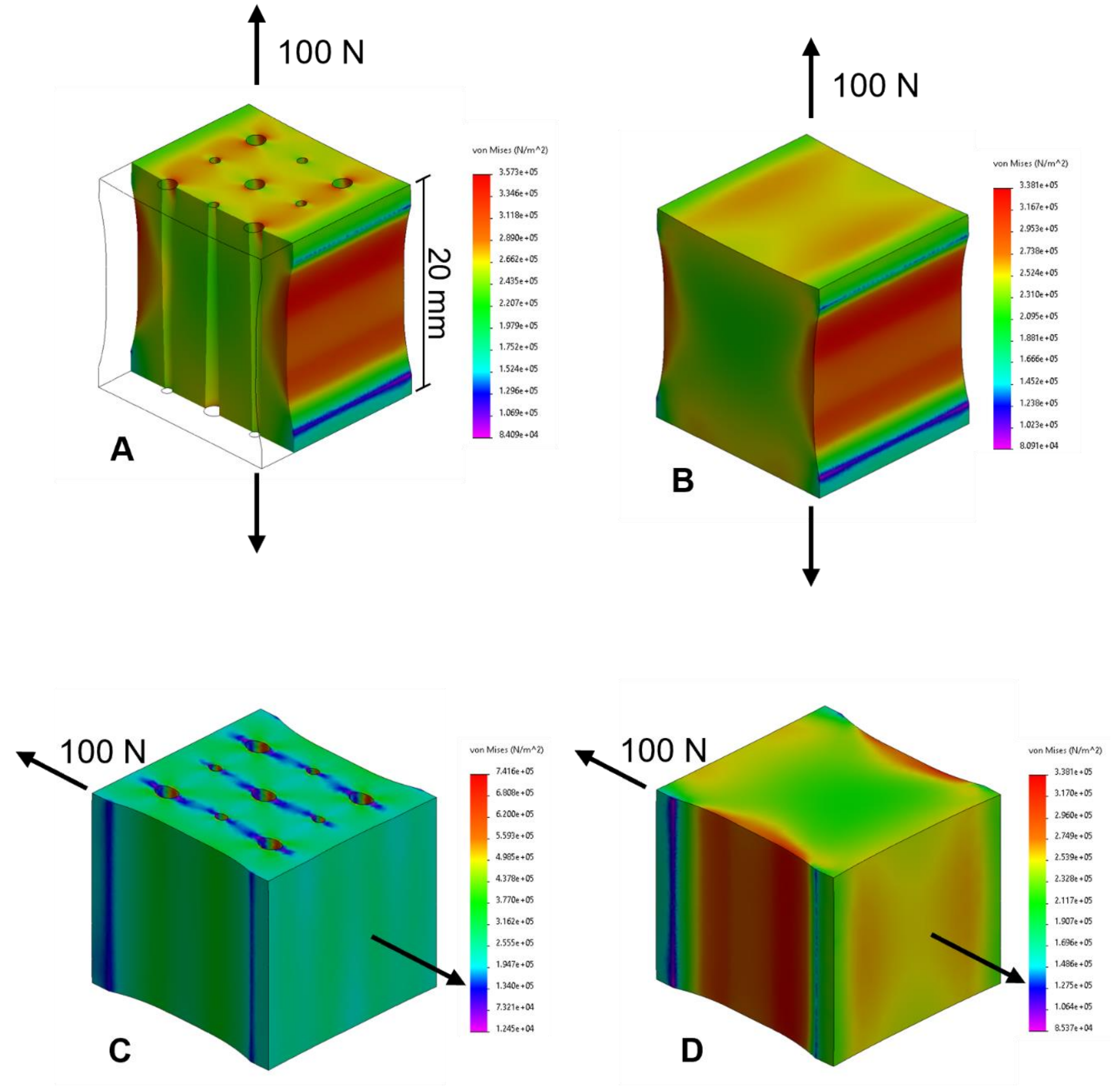

Figure S22. Simple FEA simulations of a 20 mm cubic sample loaded in tension with a force of 100 N to indicate the increase in stress due to the addition of *Injections*. Sample geometry includes 1 mm per side tensile sample shoulders. (A) Sample with *Injections* channels array showing max stress of 3.6 x 10^5 N.mm^{-2}. (B) Sample without *Injections* channels showing max stress of 3.4 x 10^5 N.mm^{-2}. (C) Sample with *Injections* channels, loaded perpendicular to the *Injections* channels, showing a max stress of 7.4 x 10^5 N.mm^{-2}. (D) Sample without Injections channels, loaded perpendicular to the *Injections* channels, showing a max stress of 3.4 x 10^5 N.mm^{-2}. Simulations performed in Solidworks Simulation (V2021, Dassault Systems, Vélizy-Villacoublay, France). Default solver settings were applied, 0.4 – 1.5 mm mesh element size and PA6 was used as the sample material.

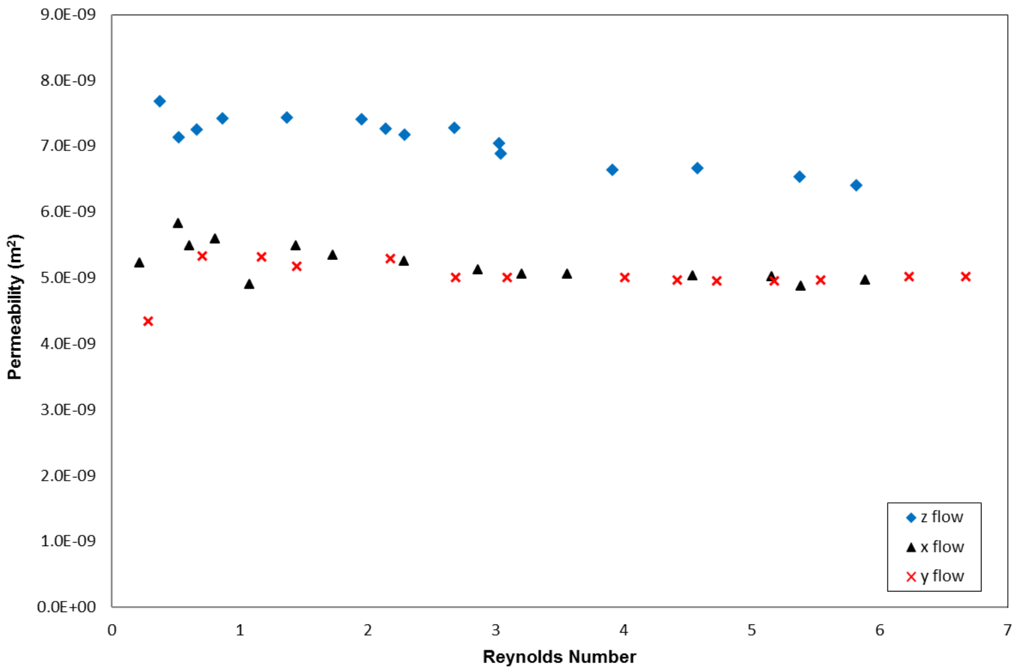

Figure S23. Intrinsic permeability plotted against Reynolds number calculated from the experimental measurements of pressure drop vs average velocity for flow through the X, Y and Z scaffold samples. Constant values of permeability with changing Reynolds number indicate Darcian flow where there is a linear relationship between pressure drop and average fluid velocity.

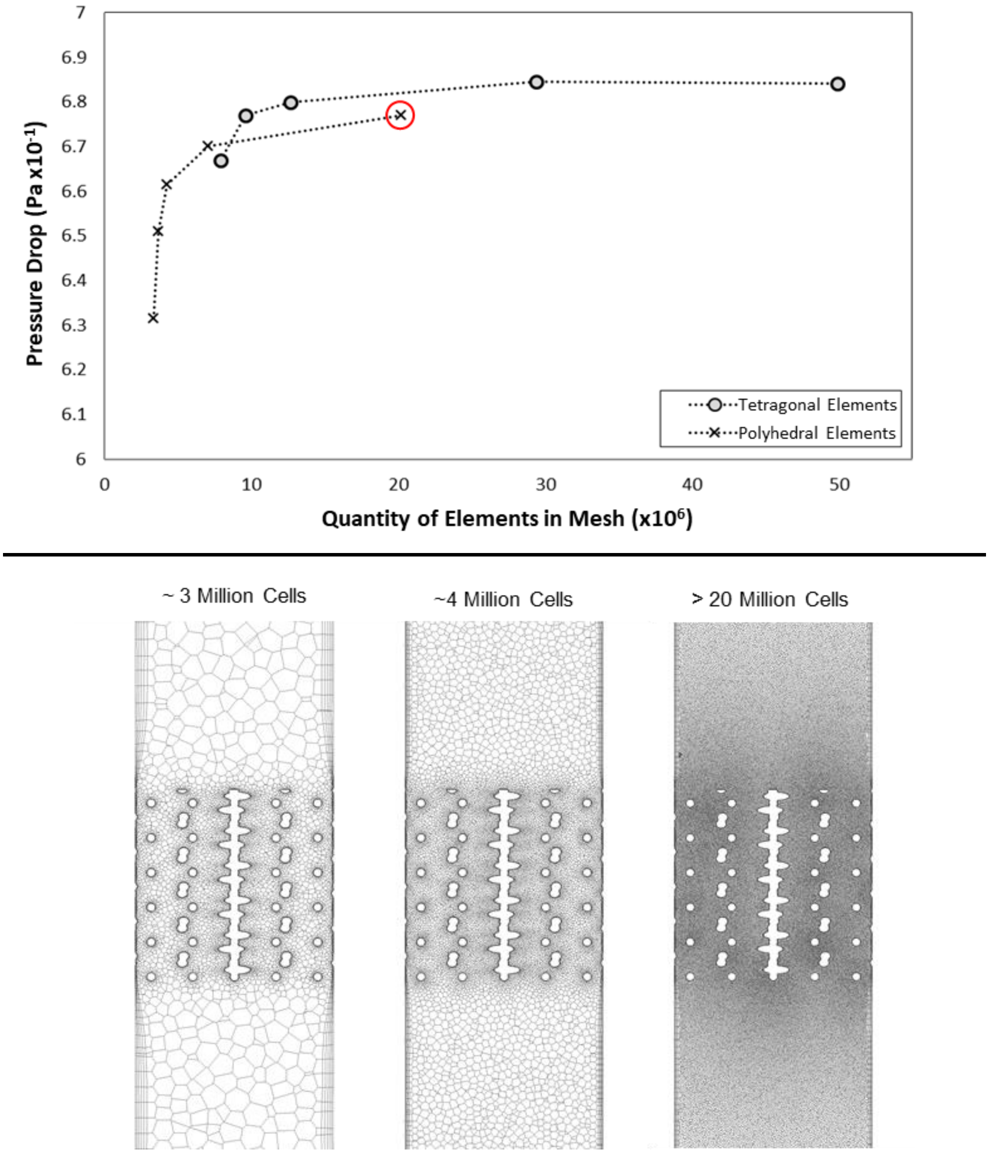

Figure S24. (top) Mesh independence results for Z flow orientation CFD simulation with CAD geometry, where the 20 million polyhedral elements setting was selected for the microscale fluid dynamics simulations. (bottom) Example meshing images for polyhedral elements.

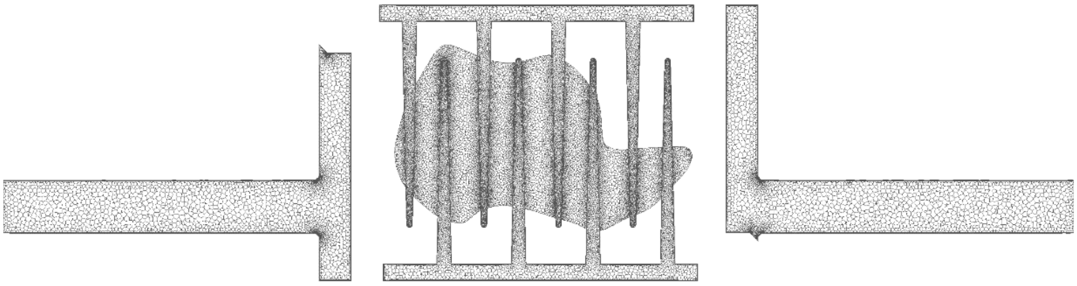

Figure S25. Moderate mesh refinement of the ***injections*** method macro-scale simulation with polyhedral elements (**Table S3**) (9.9 million elements).

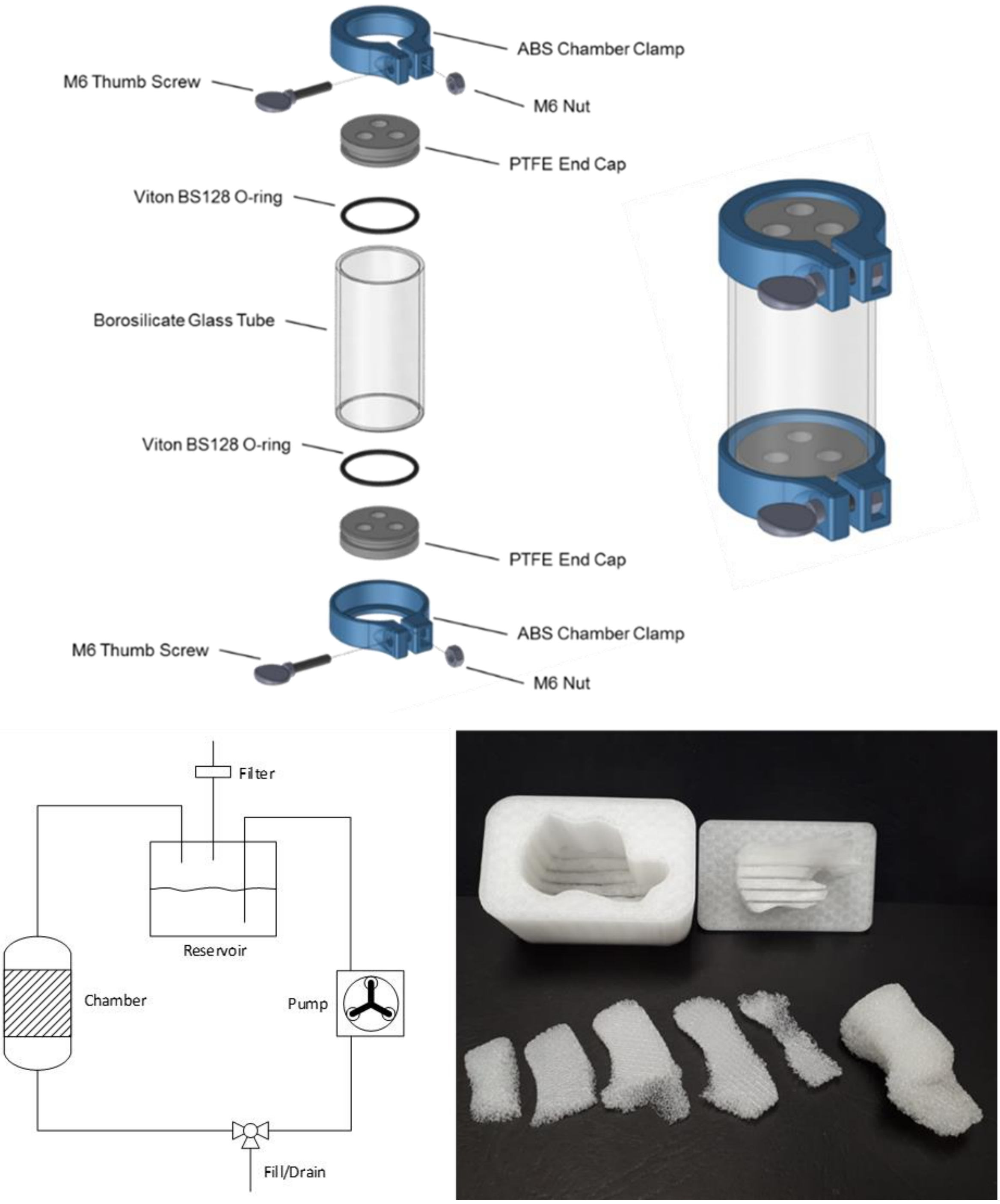

Figure S26. (Top) Bioreactor chamber assembly. (Bottom Left) A bioreactor system schematic including chambers, flow delivery, pump and mount to be placed in the incubator. (Bottom Right) Scaffold 3D printed sectioning guides alongside sectioned scaffold slices.

Table S1. Summary of viscosity properties used in previous perfusion cell culture studies.

Reference	Study	Viscosity mPa.s (dynamic)	Comments
[43]	CFD compared with experimental measurements	No viscosity value reported	
[68]	Flow simulation in a bioreactor design; no experimental study	0.89	Source of viscosity value not mentioned
[39]	Experimental water flow through scaffold compared to CFD	1	Water at 21 °C
[34]	CFD modelling of perfusion bioreactor concept design; no experimental study	0.7	Source of viscosity value not mentioned
[40]	Scaffold permeability prediction from CAD and µCT data. Experimental check using water flow	1	Water at 21 °C was assumed to be equivalent to culture media.
[37]	Wall shear stress and flow through µCT reconstructed geometry	0.851	Source of viscosity value not mentioned
[36]	Oxygen and shear stress in perfusion system, experimental comparison included	0.81	Value taken from previous study, although is slightly different compared to the reference[38]
[69]	Flow modelling in conical bioreactor compared with PIV	No viscosity value reported	
[70]	Wall shear stress and flow prediction, no experimental comparison	0.82	Value taken from previous study in literature[38]
[71]	Wall shear stress and velocity through µCT reconstructed scaffold geometry with no experimental comparison	0.81	Value taken from previous study, although is slightly different compared to the reference[38]
[28]	Perfusion bioreactor with diffuser culture experiment and CFD for added information. Shear stresses investigated	No viscosity value reported	
[72]	Fluid field properties such as velocity and shear stress through µCT scaffold geometry	0.15 length unit/time step^2 (kinematic)	Viscosity of culture media measured. Alpha MEM, no additional supplements reported
[73]	PIV and prototype bioreactor CFD and experimental comparison	1.00013	Value taken from previous literature[74]
[38]	CFD study results compared against cell culture results. Flow induced shear stress effects on cells	0.82	Measured DMEM with a number of supplements, measured at 37°C

Table S2. Summary of perfusion culture studies reporting positive cell proliferation rates.

Study	Flow Rate per million Cells (mL/min.10^6cells)
[63]	0.86
[19]	0.26
[64]	2.25
[65]	0.46
[66]	0.13
[25]	0.75
Average	0.78

Table S3. Permeability properties assigned to scaffold and diffuser insert zones within the macro CFD simulations.

Scaffold Micro Study Orientation	Scaffold Macro CFD Orientation	Permeability m²	Viscous Resistance (1/m²)
Z flow	Y Direction	7.30E-09	1.37E+08
Y flow	X Direction	5.34E-09	1.87E+08
X flow	Z Direction	5.34E-09	1.87E+08

Table S4. Macroscale CFD mesh independence study for all bioreactor designs. Bold rows indicate settings for final results.

Cell Type	Mesh size (Cells x 10^6)	Inlet pressure (Pa)	Pressure Difference Compared to Largest Mesh	Mesh Size Compared to Largest Mesh
Open chamber	0.4	4.86	0.0%	2%
	0.5	4.86	0.1%	2%
	0.5	4.86	0.0%	2%
	1.8	4.86	0.2%	8%
	**3.6**	**4.86**	**0.2%**	**16%**
	22.9	4.87	0.0%	100%
Cassette	0.2	12.26	0.5%	7%
	0.4	12.29	0.3%	14%
	1.3	12.32	0.1%	42%
	**3.0**	**12.33**	**0.0%**	**100%**
Diffuser	0.4	12.96	0.6%	8%
	0.5	12.97	0.6%	11%
	**4.5**	**13.04**	**0.0%**	**100%**
Injections	7.9	4.34	0.6%	63%
	9.9	4.36	0.2%	79%
	**12.5**	**4.37**	**0.0%**	**100%**

Table S5. Short Term Perfusion Experimental Conditions

**QTY Seeded Cells per scaffold (x10^6 cells)**	7.6
**Culture Duration (days)**	7
**Experimental Conditions**	Day 1 - Seed Cells<br>Day 1.5 - Seeding result & flow on 28 mL/min<br>Day 4 - Flow increased to 40 mL/min<br>Day 5 – Flow 40 mL/min<br>Day 8 – Results processed
**Samples**	*Seed Replicate 1*<br>*Seed Replicate 2*<br>*Open chamber*<br>*Cassette*<br>*Diffuser*<br>*Injections*
**Total Scaffolds**	6

Table S6. Long Term Perfusion Experimental Conditions.

	**20-Day Culture**	**24-Day Culture**
**QTY Seeded Cells (x10^6 cells)**	1.6	3
**Culture Duration (days)**	20	24
**Experimental Conditions**	Day 1 - Seed<br>Day 2 - Seeding result<br>Day 3 - Load perfusion chambers<br>Day 4 - Flow on 14 mL/min<br>Day 5 - Flow Increased 30 mL/min<br>Day 6 – Intermittent flow 20 mL/min [12 min on/ 60 min off]<br>Day 13 - Constant flow 20 mL/min<br>Day 15 - Flow increased 40 mL/min<br>Day 20 - Results processed	Day 1 - Seed<br>Day 2 - Seeding result<br>Day 3 - Load perfusion chambers<br>Day 4 – Flow on 14 mL/min<br>Day 7 – Flow Increased 30 mL/min<br>Day 8 – Intermittent flow 20 mL/min [12 min on/ 60 min off]<br>Day 15 – Constant flow 20 mL/min<br>Day 17 - Flow increased 40 mL/min<br>Day 24 – Results processed
**Samples**	*Seed*<br>*Static*<br>*Open chamber*<br>*Cassette*<br>*Diffuser*<br>*Injections*	*Seed*<br>*Static*<br>*Open chamber*<br>*Cassette*<br>*Diffuser*<br>*Injections Replicate 1*<br>*Injections Replicate 2*
**Total Scaffolds**	6	7
